# Super-enhancer-associated MEIS1 promotes transcriptional dysregulation in Ewing sarcoma in co-operation with EWS-FLI1

**DOI:** 10.1093/nar/gky1207

**Published:** 2018-11-28

**Authors:** Lehang Lin, Moli Huang, Xianping Shi, Anand Mayakonda, Kaishun Hu, Yan-Yi Jiang, Xiao Guo, Li Chen, Brendan Pang, Ngan Doan, Jonathan W Said, Jianjun Xie, Sigal Gery, Xu Cheng, Zhaoyu Lin, Jinsong Li, Benjamin P Berman, Dong Yin, De-Chen Lin, H Phillip Koeffler

**Affiliations:** 1 Guangdong Provincial Key Laboratory of Malignant Tumor Epigenetics and Gene Regulation, Medical Research Center, Sun Yat-Sen Memorial Hospital, Sun Yat-Sen University, Guangzhou 510120, P.R. China; 2 Department of Medicine, Cedars-Sinai Medical Center, Los Angeles, CA 90048, USA; 3 School of Biology and Basic Medical Sciences, Soochow University, Suzhou 215123, P.R. China; 4 Cancer Science Institute of Singapore, National University of Singapore, 117599, Singapore; 5 Department of Pathology, National University Hospital Singapore, 119074, Singapore; 6 Department of Pathology and Laboratory Medicine, University of California Los Angeles and David Geffen School of Medicine, Los Angeles, CA 90095, USA; 7 Department of Biochemistry and Molecular Biology, Medical College of Shantou University, Shantou 515041, P.R. China; 8 Department of Oral & Maxillofacial Surgery, Sun Yat-Sen Memorial Hospital, Sun Yat-Sen University, Guangzhou 510120, P.R. China; 9 Department of Bioinformatics and Functional Genomics, Cedars-Sinai Medical Center, Los Angeles, CA 90048, USA; 10 National University Cancer Institute, National University Hospital Singapore, 119074, Singapore

## Abstract

As the second most common malignant bone tumor in children and adolescents, Ewing sarcoma is initiated and exacerbated by a chimeric oncoprotein, most commonly, EWS-FLI1. In this study, we apply epigenomic analysis to characterize the transcription dysregulation in this cancer, focusing on the investigation of super-enhancer and its associated transcriptional regulatory mechanisms. We demonstrate that super-enhancer-associated transcripts are significantly enriched in EWS-FLI1 target genes, contribute to the aberrant transcriptional network of the disease, and mediate the exceptional sensitivity of Ewing sarcoma to transcriptional inhibition. Through integrative analysis, we identify MEIS1 as a super-enhancer-driven oncogene, which co-operates with EWS-FLI1 in transcriptional regulation, and plays a key pro-survival role in Ewing sarcoma. Moreover, APCDD1, another super-enhancer-associated gene, acting as a downstream target of both MEIS1 and EWS-FLI1, is also characterized as a novel tumor-promoting factor in this malignancy. These data delineate super-enhancer-mediated transcriptional deregulation in Ewing sarcoma, and uncover numerous candidate oncogenes which can be exploited for further understanding of the molecular pathogenesis for this disease.

## INTRODUCTION

Ewing sarcoma is the second most common and devastating primary bone malignancy that arises predominantly in children and adolescents. Despite progress in its treatment over the past decades, the survival rate of its patients is not satisfactory, pointing to the urgent need for more effective therapies centered on the molecular basis of the cancer (1–3).

Recent studies have highlighted that the disease-defining fusion protein EWS-FLI1 utilizes directly divergent chromatin remodeling mechanisms to either activate or repress enhancer elements in Ewing sarcoma (4,5). Therefore, characterization of epigenomic deregulation in Ewing sarcoma may provide innovative insights into the pathophysiology of this cancer and offer new therapeutic approaches.

Interestingly, studies have noted that relative to typical enhancers, transcripts associated with super-enhancers are enriched for active EWS-FLI1 binding motifs (6). By regulating cell-type specific genes, super-enhancer is a large cluster of cis-regulatory DNA elements densely bound by transcription factors and cofactors, playing critical roles in defining cell fate and identity (7,8). Histone marks such as H3K27ac, H3K4me1 and the transcription cofactor p300 often are used to define super-enhancers (4,6,9–11). Importantly, super-enhancers frequently drive high-level expression of prominent oncogenes in cancer cells. In particular, we and others have recently shown that the expression of super-enhancer-associated genes is disproportionately vulnerable to transcriptional perturbation, mediating the addiction of cancer cells to high-level transcription (10–14).

In the current study, we observed that Ewing sarcoma cells were sensitive to transcriptional perturbation, mediated by CDK7 suppression. Analyses of four cell lines and three primary tumors established the super-enhancer landscape of Ewing sarcoma, and revealed that super-enhancer-associated genes were exceptionally sensitive to inhibition of CDK7. Further investigation based on the biological attributes of super-enhancers identified MEIS1 as a novel oncogenic transcription factor that played a key pro-survival role in Ewing sarcoma, via activating the transcription of APCDD1 cooperatively with EWS-FLI1.

## MATERIALS AND METHODS

### Cell culture

Ewing sarcoma cell lines (A673, SKNMC, TC32, TC71, EW8, TCC446 and EWS502) used in this study were described previously (15,16). Briefly, they were grown in Dulbecco's modified Eagle's medium (DMEM) containing 10% fetal bovine serum (FBS) and 1% penicillin–streptomycin, and kept at 37°C with 5% CO_2_. The identity of all cell lines was recently authenticated by short tandem repeat analysis. All cells were tested to be free of mycoplasma contamination.

### Antibodies, reagents and kits

The following antibodies were used in the current study: CDK7 (Cell Signaling Technology, 2916), RNAPII CTD S2 (Bethyl, A300–654A), RNAPII CTD S5 (Bethyl, A300–655A), RNAPII CTD S7 (Cell Signaling Technology, 13780), RNAPII (Santa Cruz, sc-899), MEIS1 (Abcam, ab19867), APCDD1 (Novus Biologicals, NB110–92756SS), FLI-1 (Santa Cruz Biotechnology, sc-53826), GAPDH (Cell Signaling Technology, 5174), anti-mouse IgG-HRP (Santa Cruz Biotechnology, sc-2005), anti-rabbit IgG-HRP (Santa Cruz Biotechnology, sc-2004) and rabbit IgG Isotype Control (Invitrogen, 02–6102). Reagents and kits included: THZ1 (ApexBio, A8882), FITC Annexin V Apoptosis Detection Kit (BD Biosciences), Dual-Luciferase Reporter Assay System (Promega), BioT transfection reagent (Bioland Scientific), Lipofectamine 2000 transfection reagent (Invitrogen), Lipofectamine RNAiMAX transfection reagent (Invitrogen), and siRNA pools targeting MEIS1 and APCDD1 (Dharmacon, Supplementary Table S1).

### MTT cell proliferation assay

Cells were seeded into 96-well plates at a density of 3000–4000 cells per well and cultured for indicated time course. For THZ1 treatment, the cultural medium was replaced with fresh complete medium containing either DMSO or THZ1 at day 0 once the cells were attached to the plates. Cell proliferation was measured by standard MTT assay as described previously (17).

### Apoptosis assay

Cell apoptosis was measured using flow cytometric analysis of double staining with Annexin V and propidium iodide (PI) according to the manufacturer's instructions (BD Biosciences). Data were analyzed using FlowJo 7.6 software (Tree Star).

### Soft agar colony formation assay

Soft agar colony formation assay was performed to evaluate the anchorage independent growth of Ewing sarcoma cells. Briefly, a bottom layer solution (0.6% agarose in DMEM with 10% FBS) was added into 12-well plate and solidified before use. 1500 cells were then mixed with 500 μl of top layer solution (0.3% low melting agarose in DMEM with 10% FBS) and spread over the bottom layer. After solidified at 4°C, 0.5 ml of feeder medium was added into each well. The plates were kept in a 5% CO_2_ incubator at 37°C for 2 weeks. Colonies were stained with 0.01% crystal violet in 4% paraformaldehyde/PBS and quantified using ImageJ software.

### Chromatin immunoprecipitation (ChIP) assay

To cross-link DNA and protein, 2 × 10^7^ of A673 cells were fixed with 1% formaldehyde for 10 min at room temperature. Chromatin solution was prepared following a standard protocol (10). For immunoprecipitation, solubilized chromatin was incubated with 5μg of anti-MEIS1 antibody, anti-FLI-1 antibody, or IgG control overnight at 4°C on a rotating wheel. Antibody-chromatin complexes were subsequently pulled down by incubating with Dynabeads Protein G (Life Technologies) for 4 h at 4°C. After reversal of crosslink, RNase A as well as Proteinase K treatment, immunoprecipitated DNA was extracted with the Min-Elute PCR purification kit (Qiagen), followed by qPCR analysis, or DNA library preparation and sequencing on HiSeq 3000 platform. Primers for qPCR analysis are listed in Supplementary Table S2.

### qRT-PCR

Total RNA was extracted using RNeasy mini kit (Qiagen), and 1 μg aliquots were used for cDNA synthesis using the qScript™ cDNA Synthesis Kit (Quanta Biosciences). The cDNA templates were subjected to PCR amplification on CFX96 qPCR System (Biorad). Expression of each gene was normalized to GAPDH, and quantified using 2^−delta(ct)^ method. Primers are listed in Supplementary Table S3.

### Immunoblotting assay

Cells were lysed in RIPA lysis and extraction buffer (Thermo Fisher Scientific), supplemented with proteinase inhibitor cocktail and phosphatase inhibitor cocktail (Roche) for 30 min on ice. Protein quantification was determined by Bradford assay. Immunoblotting was performed using SDS-PAGE followed by protein transfer to PVDF membrane (Bio-Rad). Primary antibodies were incubated overnight in cold room. Secondary antibodies were incubated for 1–2 h at room temperature. Indicated antibodies are listed above.

### Retroviral and lentiviral infections

Retroviral MSCV-PIG-MEIS1 plasmid and MSCV-PIG control vector were generous gifts from Dr. Jianjun Chen (University of Cincinnati, College of Medicine). Lentiviral pLVX-DsRed-APCDD1 plasmid, and pLKO.1-puro or pLKO-Tet-On vector based shRNAs targeting CDK7, EWS-FLI1, MEIS1 and APCDD1 were constructed and confirmed by DNA sequencing. All retroviral and lentiviral particles were prepared in HEK293T cells. For retroviral particle preparation, MSCV-PIG-MEIS1 and pCL-Eco packaging vector were co-transfected using BioT transfection reagent. For lentiviral particle production, recombinant vectors were co-transfected with packaging vectors (psPAX2 and pMD2.G). Supernatants containing viral particles were harvested 24, 48 and 72 h after transfection and filtered (pore size: 0.45 μm). To generate stable cell lines, Ewing sarcoma cells were transduced with virus-containing medium in the presence of 8μg/mL polybrene for 16 hours, and selected in 1μg/ml puromycin for 48 h post-infection. shRNA target sequences are listed in Supplementary Table S4.

### Luciferase reporter assay

Super-enhancer constituent elements (∼500 bp) were cloned into the Firefly luciferase reporter vector pGL3-Promoter (Promega). A673 and SKNMC cells were transfected using Lipofectamine 2000 transfection reagent. A Renilla luciferase control plasmid was co-transfected as a normalization control. Luciferase activity was measured using the Dual-Luciferase Reporter Assay System (Promega). Primers used for PCR amplification of luciferase reporter vector construction are listed in Supplementary Table S5.

### Identification and analysis of super-enhancers

H3K27ac ChIP-seq raw data generated in four Ewing sarcoma cell lines and three primary tumors (4,6) were retrieved from NCBI Gene Expression Omnibus (GEO) or generously shared by Dr Kimberly Stegmaier (Harvard Medical School). Data were re-analyzed to identify super-enhancers based on the method previously described (8).

### Gene ontology (GO) and gene set enrichment analysis (GSEA)

GO analysis was performed using consensusPath DB (cpdb.molgen.mpg.de). Significant enriched biological processes were defined as *P* < 0.01. GSEA for super-enhancer- and typical-enhancer-associated transcripts of Ewing sarcoma was performed as previously described (10).

### Motif analysis

DNA segments predicted to be bound by MEIS1 in Ewing sarcoma were extracted for motif analysis by Homer (http://homer.ucsd.edu/homer/motif/). Homer script findMotifsGenome.pl was run on all segments with augment ‘hg19-size200-len10-noMotif’to detect enrichment of known transcription factor motifs. Homer results were further manually examined, and those motifs with high background enrichment were removed.

## RESULTS

### THZ1 exerts potent anti-neoplastic effect in Ewing sarcoma

**Figure 1.**
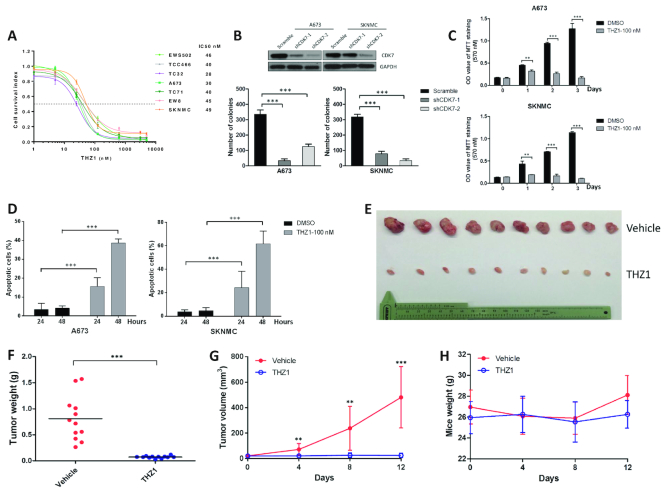
THZ1 exhibits strong anti-neoplastic property against Ewing sarcoma. (**A**) Dose-response curves of 7 Ewing sarcoma cell lines to THZ1 treatment for 72 hr. IC50 values were measured by MTT assay. Data were represented as mean ± SD of three replicates. (**B**) A673 and SKMNC cells stably expressing either CDK7-specific shRNAs or scrambled shRNA control (Scramble) were subjected to immunoblotting assay (upper panel) and colony formation assay (lower panel). Bars represent mean ± SD of three replicates (*** *P* < 0.001). (**C**) MTT assay and (**D**) apoptosis assay showing the effect of THZ1 treatment on Ewing sarcoma cell lines at indicated time points. Bars represent mean ± SD of three replicates (**P < 0.01, ***P < 0.001). (E–G) THZ1 suppressed the growth of A673 xenografts. (**E**) Images and (**F**) weights of resected tumors from both vehicle and THZ1 treatment groups at the end point. (**G**) Tumor volumes of mice treated either with vehicle or THZ1. Data represent mean ± SD of each group (**P < 0.01, ***P < 0.001). (**H**) No significant loss of body weight was observed in mice during treatment.

Our recent work in other cancer types demonstrated that inhibiting transcriptional process can be exploited as a potential therapeutic strategy (10,11). Given that transcriptional dysregulation typifies the biology of Ewing sarcoma, we first evaluated the anti-tumor effect of transcription inhibition in Ewing sarcoma cells by testing THZ1, which is a newly developed covalent inhibitor of cyclin-dependent kinase 7 (CDK7). We and others have recently reported the growth inhibitory effects of THZ1 in multiple types of cancers via suppressing CDK7-dependent transcriptional activation (10–14,18). Dose-response experiments showed that all seven representative Ewing sarcoma cell lines were highly sensitive to THZ1 treatment, with IC_50_ values ranging from 28 to 49 nM (Figure 1A). In fact, these IC50 values in Ewing sarcoma cells were lower than the majority of solid tumor cells tested in the same setting (10,11,13,14,18). Depletion of CDK7 expression by shRNAs confirmed that CDK7 was indispensable for the viability of Ewing sarcoma cells (Figure 1B).

In vitro proliferation and apoptosis assays subsequently showed that THZ1 treatment led to profound reduction of proliferation and massive induction of apoptosis in Ewing sarcoma cells (Figure 1C, D). We next tested the anti-tumor effect of THZ1 in NOD scid gamma (NSG) murine model, where each mouse carried two explants formed by A673 cells. Mice bearing tumors were randomly divided into two groups and treated with either vehicle or THZ1 twice daily (10 mg/kg). Strikingly, THZ1 treatment completely abolished tumor growth *in vivo*, highlighting the strong anti-Ewing sarcoma property of this compound (Figure 1E–G). Importantly, in these experimental mice, neither significant loss of body weight (Figure 1H) nor other common toxic effects (e.g. diarrhea, rash, etc. data not shown) were observed, in agreement with our previous reports (10,11).

### CDK7 inhibition elicits selective transcriptional repression in Ewing sarcoma cells

**Figure 2.**
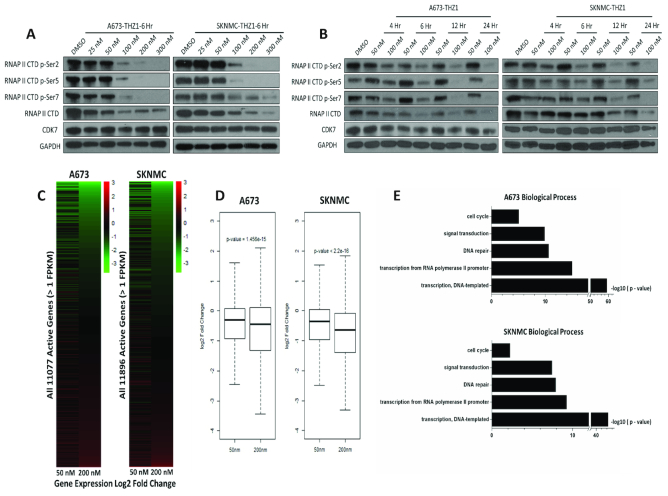
THZ1 selectively inhibits RNAPII-mediated transcription in Ewing sarcoma, (**A**, **B**) Immunoblotting analyses of RNAP II CTD phosphorylation in A673 and SKMNC cells treated either with THZ1 or DMSO at the indicated concentrations for the indicated durations. (**C**) Heatmap and (**D**) Box plots showing changes in gene expression in A673 and SKNMC cells following treatment with either 50 or 200 nM THZ1 for 6 h. (**E**) Selected Gene ontology (GO) functional categories of transcripts decreased over 2-fold in A673 and SKNMC cells following 50 nM THZ1 treatment for 6 h.

We next probed the mechanisms underlying the sensitivity of Ewing sarcoma cells to THZ1. CDK7 phosphorylates RNA Polymerase II C-terminal domain (RNAP IICTD), thereby regulating transcriptional initiation and pause release, as well as elongation (19–21). Accordingly, we observed a dose- and time-dependent decrease in RNAP IICTD phosphorylation at both initiation-associated serine 5 (S5) and serine 7 (S7) as well as the elongation-associated serine 2 (S2) in both A673 and SKNMC cells upon THZ1 treatment (Figure 2A, B).

The THZ1-induced transcriptional alterations in Ewing sarcoma cells were further studied using whole-transcriptome sequencing (RNA-Seq) in both A673 and SKNMC cell lines, following treatment with 50 and 200 nM THZ1 for 6 hours. These concentrations and time points were selected because they represented either limited (50 nM, low-dose) or substantial (200 nM, high-dose) inhibition of RNAP IICTD phosphorylation (Figure 2A, B). Of note, compared to high-dose treatment, low-dose THZ1 elicited transcriptional inhibition in a more selective manner (Figure 2C, D; Supplementary Dataset S1). To begin to understand the biological characteristics of transcripts which were particularly sensitive to THZ1, Gene ontology (GO) analysis was performed and those THZ1-sensitive transcripts showed significant enrichment in cellular processes including transcriptional regulation, DNA repair, signal transduction and cell cycle regulation (Figure 2E). These results highlighted the prominent roles of these THZ1-sensitive transcripts in Ewing sarcoma, and suggested that they might be pivotal in mediating the exceptional sensitivity of Ewing sarcoma cells to CDK7 inhibition.

### Characterization of super-enhancer landscape in Ewing sarcoma

Previous work including ours has demonstrated that expression of super-enhancer-associated genes requires persistent transcriptional activation; and thus, they are particularly vulnerable to transcriptional inhibition (10–14,18). Therefore, super-enhancers in Ewing sarcoma may provide mechanistic explanation for THZ1-mediated selective transcription inhibition.

**Figure 3.**
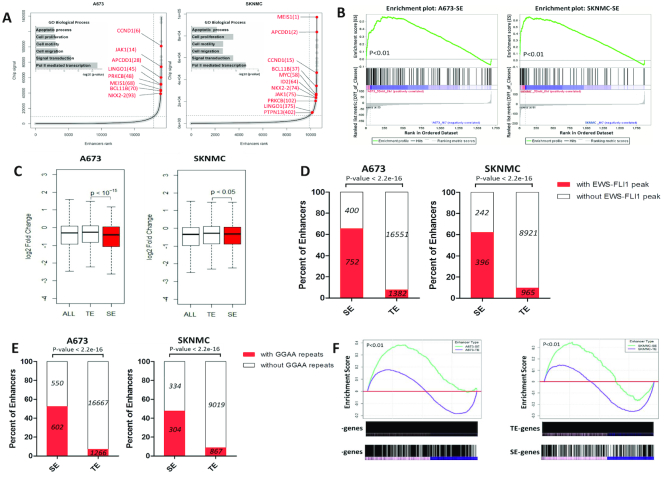
Profiling of super-enhancer landscapes in Ewing sarcoma cell lines, (**A**) Hockey stick plots showing rank order of H3K27ac signals for all enhancers in A673 and SKNMC cells. Inserted panels showing selected GO functional categories of super-enhancer-associated genes. (**B**) Gene Set Enrichment analysis (GSEA) of the fold changes of super-enhancer (SE)-associated transcripts following treatment with 50 nM THZ1 for 6 h in A673 and SKNMC. (**C**) Box plots showing log2 fold changes of transcripts associated with typical-enhancer (TE) and SE upon 50 nM THZ1 treatment for 6 h in A673 and SKNMC. (D, E) Pearson's Chi-squared tests showed SE regions had significantly more (**D**) EWS-FLI1 binding peaks and (**E**) motif (GGAA repeats) than TE regions. EWS-FLI1 ChIP-seq data are publicly accessible on NCBI GEO under the accession number ‘GSE61953′. (**F**) GSEA of the fold changes of either SE- or TE-associated transcripts upon EWS-FLI1 depletion (48 hr) in A673 and SKNMC cells. Gene expression data for A673 and SKNMC cells in either the presence or absence of EWS-FLI1 knockdown were also obtained from NCBI GEO (GSE61953).

To this end, we analyzed available H3K27ac ChIP-seq data generated in 4 Ewing sarcoma cell lines and 3 primary tumors (4,6), and identified super-enhancer-assigned genes (Figure 3A; Supplementary Figure S1 and Datasets S2–8). GO analysis revealed that super-enhancer-assigned genes were significantly enriched in cellular processes representing ‘cancer hallmarks’ (22), such as cell proliferation, apoptosis, motility and migration (Figure 3A; Supplementary Figure S1). Importantly, Gene Set Enrichment analysis (GSEA) showed that THZ1-sensitive transcripts were significantly enriched in the gene sets associated with super-enhancers, but not typical-enhancers (Figure 3B). Moreover, preferential downregulation of transcripts associated with super-enhancers, but not typical-enhancers, was observed upon THZ1 treatment (Figure 3C).

Super-enhancer elements are frequently associated with and regulated by master transcription factors in normal cell development (8,23). Since the disease-defining fusion gene EWS-FLI1 orchestrates the transcriptional dysregulation in Ewing sarcoma, we hypothesized that EWS-FLI1 also dominates the regulation of super-enhancers in Ewing sarcoma. To this end, we used Pearson's Chi-squared test to compare the proportions of super-enhancer regions to typical-enhancer regions containing EWS-FLI1 binding peaks. Importantly, EWS-FLI1 peaks fell significantly more within super-enhancer regions in both A673 and SKNMC cell lines (Figure 3D). Moreover, GGAA repeats, the established active EWS-FLI1 binding motif in Ewing sarcoma (4,6,24,25), were enriched in a much higher degree in super-enhancer regions relative to typical-enhancer ones (Figure 3E). Importantly, we further performed GSEA in A673 and SKNMC cells to interrogate gene expression profiles in either the absence or presence of EWS-FLI1 knockdown, and found that super-enhancer-associated transcripts were significantly enriched among genes whose expressions were altered by EWS-FLI1, compared with typical-enhancer-associated transcripts (Figure 3F).

These results suggested that partially under the regulation of EWS-FLI1, super-enhancers mediate the superior vulnerability of Ewing sarcoma cells to CDK7 inhibition, and play key roles in the biology of Ewing sarcoma.

### Identification of MEIS1 as a super-enhancer-associated gene in Ewing sarcoma

**Figure 4.**
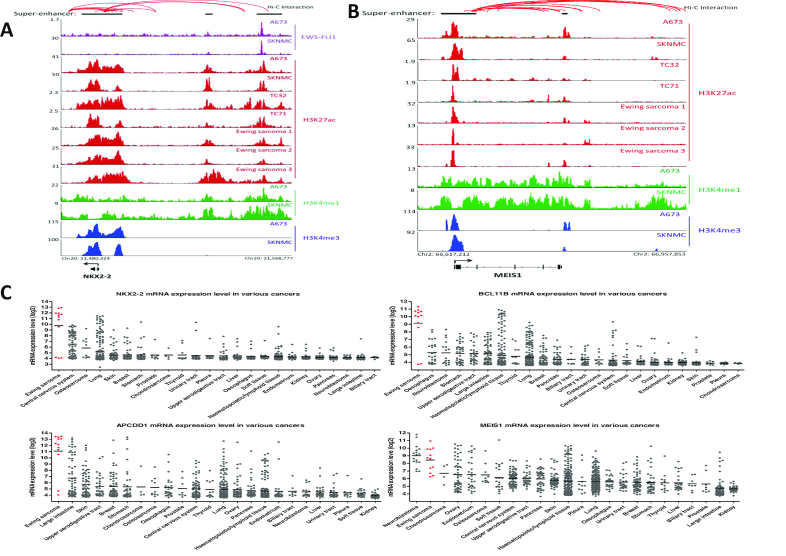
Nomination of super-enhancer-associated transcripts in Ewing sarcoma, (**A**, **B**) ChIP-seq profiles of EWS-FLI1, H3K27ac, H3K4me1 and H3K4me3 at representative super-enhancer-associated gene loci across Ewing sarcoma cell lines and primary tumors. Y axis represents the value of reads per million per base pair (rpm/bp). Above the ChIP-seq profiles were interactions among cis-regulatory elements in SKNMC cells predicted by Hi-C from ENCODE project, and visualized using public browser (http://promoter.bx.psu.edu/hi-c/index.html). (**C**) Data retrieved from CCLE project depicting mRNA expression of representative super-enhancer-associated transcripts across various types of human cancer cells.

In an attempt to screen robustly for super-enhancer-associated genes functionally relevant in Ewing sarcoma biology, we required them to occur in at least three out of four Ewing sarcoma cell lines and one out of three primary samples. This approach generated a total of 147 genes (Supplementary Table S6), containing a number of known Ewing sarcoma oncogenes such as CCND1, NKX2–2, BCL11B and MYC (6,16,26–31) (Figure 4A; Supplementary Figure S2A). We also observed pro-growth factors implicated in other cancer types but not yet characterized in Ewing sarcoma, such as MEIS1, APCDD1 and IGF2BP1 (32–37) (Figures 4B and 6C; Supplementary Figure S2B). Importantly, enhancer profiles were highly similar between Ewing sarcoma cell lines and primary tumors, which were also supported by additional markers such as H3K4me1 (Figures 4A, B and 6C; Supplementary Figure S2A, B). By re-analyzing the publically-available Hi-C data of SKNMC cells, we confirmed extensive interactions between enhancers and promoters (indicated by red connecting lines above the ChIP-seq profiles in Figure 4A, B). Moreover, constituent elements within super-enhancer regions were also noted to have frequent interactions, a characteristics of active super-enhancers that was recently reported (38). Additionally, in agreement with above analysis, occupancy of EWS-FLI1 in many of these super-enhancers was evident (e.g. NKX2–2, APCDD1, CCND1, Figures 4A and 6C; Supplementary Figure S2A). Importantly, *in silico* analysis with Cancer Cell Line Encyclopedia (CCLE) showed distinct expression patterns of many of these super-enhancer-assigned genes in Ewing sarcoma (Figure 4C; Supplementary Figure S2C), underscoring the capacity of our approach to nominate lineage-specific transcripts and potential oncogenes.

Among these 147 super-enhancer-associated transcripts, we were particularly interested in MEIS1, because the intensity of its super-enhancer ranked first in SKNMC cells (Figure 3A), and it had prominent expression profiles in Ewing sarcoma (Figure 4C).

MEIS1 is a homeodomain transcription factor extensively investigated in both normal and leukemic hematopoiesis. In acute myeloid leukemia, overexpression of MEIS1 has been consistently observed (36,39–43). Importantly, in validating MEIS1 mRNA expression (Figure 4C), protein levels of MEIS1 were similarily high in Ewing sarcoma cells, and were comparable with, or even higher than that of leukemic cells; while in breast cancer cells, expression of MEIS1 was low (Supplementary Figure S3). Using luciferase reporter assay, we noted robust activity of MEIS1 super-enhancer constituents (>1.5-fold over control, *P* < 0.01) (Supplementary Figure S4). In addition, immunoblotting confirmed high sensitivity of MEIS1 to THZ1 treatment in Ewing sarcoma (Supplementary Figure S5).

### MEIS1 is a novel oncogenic factor in Ewing sarcoma

**Figure 5.**
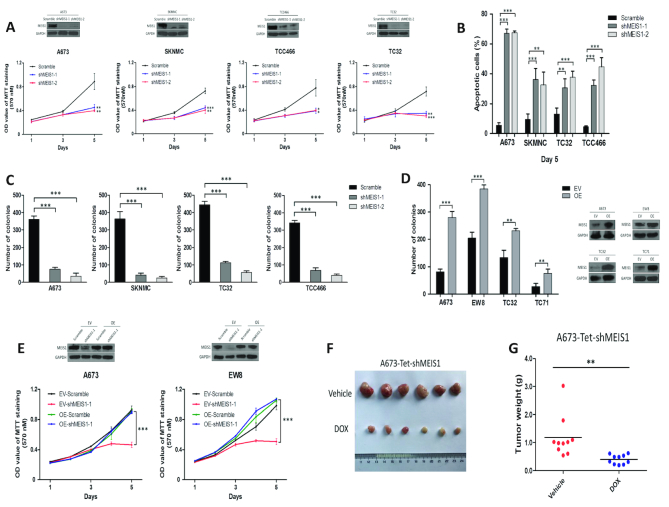
Oncogenic function of MEIS1 in Ewing sarcoma, (**A**) MTT assay, (**B**) apoptosis assay and (**C**) soft-agar assay evaluating the knockdown effects of MEIS1 on Ewing sarcoma cells. Error bars represent mean ± SD of three replicates (*P < 0.05, **P < 0.01, ***P < 0.001). Efficiency of MEIS1 knockdown in Ewing sarcoma cells was shown by immunoblotting assays. (**D**) Ewing sarcoma cells were stably transfected with either empty vector (EV) or plasmid encoding MEIS1 (OE). Immunoblotting was performed to determine MEIS1 expression, and cell anchorage-independent growth was measured by soft agar assay. Error bars represent mean ± SD of three replicates (**P < 0.01, *** *P* < 0.001). (**E**) A673 and EW8 cells stably expressing indicated plasmids were infected again with either shMEIS1 or shRNA control (Scramble), and subjected to immunoblotting and MTT assays. Bars represent mean ± SD of three replicates (***P < 0.001). (F–G) Nude mice were inoculated subcutaneously with A673 cells stably expressing inducible MEIS1 shRNA (A673-Tet-shMEIS1). Mice were randomly allocated to either doxycycline (DOX)-treated or vehicle-treated groups (*n* = 5 per group). (**F**) Representative images and (**G**) weights of resected tumors at the end point (**P < 0.01).

The biological relevance of MEIS1 in Ewing sarcoma was next assessed. Importantly, knockdown of MEIS1 by shRNAs markedly decreased cell proliferation and increased cell apoptosis (Figure 5A–C). Over-expression of MEIS1 promoted colony growth of Ewing sarcoma cells as measured by soft agar assays (Figure 5D). Furthermore, on-target effect of shRNA-mediated knockdown was confirmed by rescue assays (Figure 5E).

We next tested the dependency of Ewing sarcoma cells on MEIS1 in an in vivo setting. To this end, we generated a cell line model in which MEIS1 expression could be silenced by doxycycline (DOX)-inducible shRNA (A673-Tet-shMEIS1) (Supplementary Figure S6). Xenograft tumors were then established in nude mice by subcutaneous implantation. After inoculation, either doxycycline (25 mg/kg per day via oral gavage) or vehicle control (3% sucrose in drinking water, *ad libitum*) was administered to the mice for the duration of the experiments. Importantly, silencing of MEIS1 significantly inhibited xenograft tumor growth (Figure 5F, G; Supplementary Figure S6), corroborating the in vitro results. Collectively, these data strongly suggested that MEIS1 is a progrowth factor in Ewing sarcoma.

Further to understand the function of MEIS1 in the biology of Ewing sarcoma, ChIP-seq was performed using MEIS1 antibody in A673 cells. This generated a genome-wide occupancy profile of MEIS1 containing 1800 peaks with high confidence (Supplementary Dataset S9).

**Figure 6.**
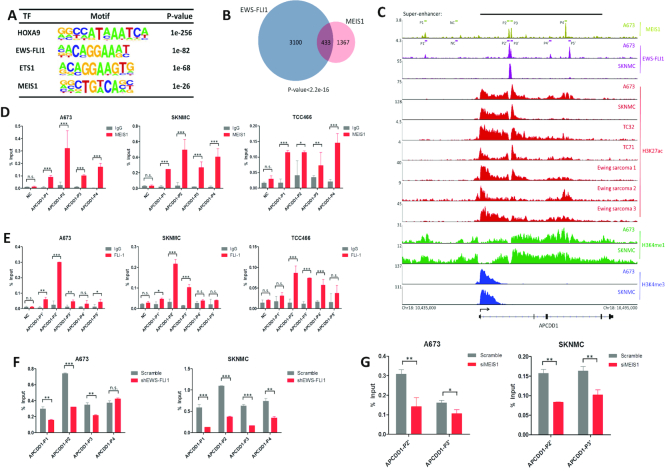
MEIS1 and EWS-FLI1 directly co-bind super-enhancer regions of APCDD1, (**A**) Homer Motif Enrichment results of known transcription factor binding motifs enriched at MEIS1 binding regions in A673 cells. (**B**) Venn diagram displaying the overlap between EWS-FLI1 and MEIS1 peaks in A673 cells. P value was calculated using Fisher's exact test. (**C**) Integrative Genomics Viewer showing MEIS1, EWS-FLI1, H3K27ac, H3K4me1 and H3K4me3 occupancy in APCDD1 gene in A673 and SKNMC cells as well as primary tumors. Olive and purple bars indicate template regions subjected to ChIP-qPCR experiments. (D-E) Confirmation of selected (**D**) MEIS1 and (**E**) EWS-FLI1 binding sites in APCDD1 locus by ChIP-qPCR. NC, negative control. Error bars represent mean ± SD of three replicates (n.s., statistically no significance, *P < 0.05, **P < 0.01, ***P < 0.001). (F–G) Either (**F**) EWS-FLI1 or (**G**) MEIS1 was silenced in A673 and SKNMC cells, and antibodies against either (**F**) MEIS1 or (**G**) FLI-1 were used to determine the transcription fator change in occupancy at selected regions of APCDD1 by ChIP-qPCR. Error bars represent mean ± SD of three replicates (n.s., statistically no significance, *P < 0.05, **P < 0.01, ***P < 0.001).

Motif analysis revealed that both MEIS1 and HOXA9 consensus motifs experimentally determined in hematopoietic cells were highly enriched in MEIS1-associated DNA fragments (Figure 6A). This result was consistent with the known functional interaction between MEIS1 and HOXA9 in hematopoietic cells, and stongly suggested the cognate recognition motifs of MEIS1 in both hematopoietic and Ewing sarcoma cells (44–46). Strikingly, the binding motif of EWS-FLI1 in Ewing sarcoma was also significantly enriched in the MEIS1-binding regions (Figure 6A).

Indeed, approximately 24% of MEIS1 binding sites (433 of 1800) overlapped with 12% of those of EWS-FLI1 (433 of 3533) (*P* < 2.2e–16, Figure 6B). Through further integration with RNA-seq data in the presence of silencing EWS-FLI1, we also found that a significant fraction (13.8%, 71 of 513) of ‘EWS-FLI1 direct target genes’ (genes bound by EWS-FLI1 and changed in expression by more than 2-fold in response to EWS-FLI1 knockdown, Supplementary Figure S7) were co-occupied by MEIS1 (Supplementary Figure S8 and Dataset S10). We validated the co-regulation of MEIS1 and EWS-FLI1 on a number of these EWS-FLI1 direct target genes by qRT-PCR (Supplementary Figure S9). Importantly, amongst these genes, there were established EWS-FLI1 downstream targets functionally important for Ewing sarcoma, such as STEAP1 and SOX5 (47,48). In addition, the co-occupancy of MEIS1 and EWS-FLI1 was notable in many of them (examples shown in Figure 6C and Supplementary Figure S10). These results indicated that MEIS1 might functionally co-operate with EWS-FLI1 in co-regulating the gene expression program in Ewing sarcoma.

### MEIS1 and EWS-FLI1 transcriptionally activate APCDD1 in Ewing sarcoma

To test further this hypothesis, focus was placed on one of their mutually targeted genes: APCDD1. APCDD1 was noted because: (i) its super-enhancer intensity ranked No.2 in SKNMC cells (Figure 3A); (ii) it was specifically upregulated in EWS cells (Figure 4C) and (iii) it has been identified as a tumor-promoting factor in colon cancer (34). Binding peaks of MEIS1 and EWS-FLI1 on APCDD1 (which were validated by ChIP-qPCR assays, Figure 6D, E) were flanked with high levels of H3K4me1 and H3K27ac modifications (Figure 6C), strongly suggesting the positive regulation of MEIS1 and EWS-FLI1 on APCDD1 transcription.

**Figure 7.**
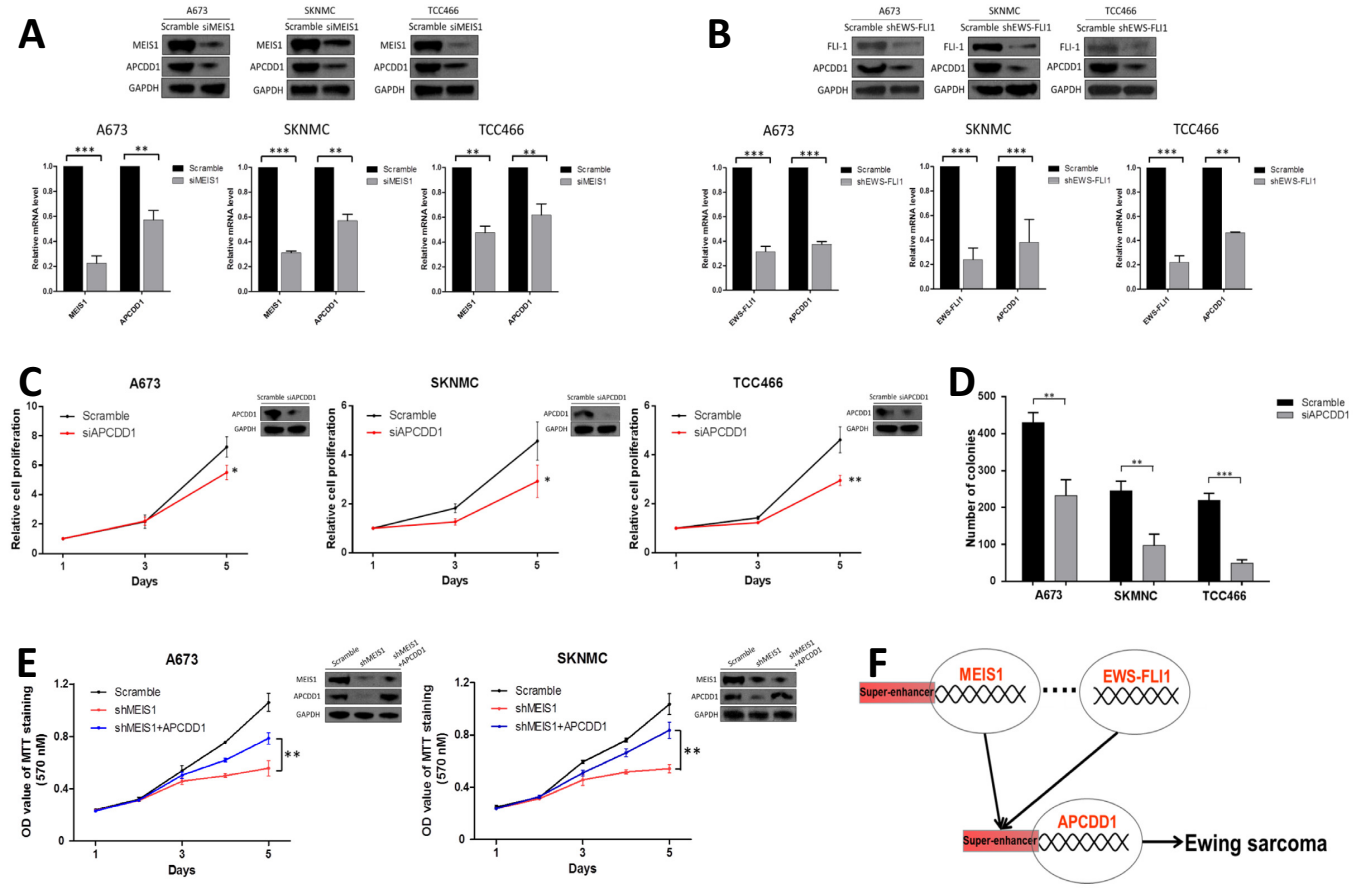
APCDD1 is co-regulated by MEIS1 and EWS-FLI1 and mediates the oncogenic role of MEIS1. (A, B) Silencing of (**A**) MEIS1 or (**B**) EWS-FLI1 downregulated expression of both APCDD1 mRNA and protein. Error bars represent mean ± SD of three replicates (**P < 0.01, ***P < 0.001). (C, D) APCDD1 knockdown potently (**C**) inhibited cell proliferation and (**D**) decreased colony formation. Error bars represent mean ± SD of three replicates (*P < 0.05, **P < 0.01, ***P < 0.001). (**E**) A673 and SKNMC cells stably expressing shMEIS1 were transiently transfected with plasmid encoding APCDD1, and subjected to immunoblotting and MTT assays. Bars represent mean ± SD of three replicates (**P < 0.01). (**F**) Proposed model showing that MEIS1 and EWS-FLI1 co-operatively activate APCDD1 transcription, thereby promoting the malignant phenotype of Ewing sarcoma cells.

To test the potential functional interplay between MEIS1 and EWS-FLI1 in the transcriptional regulation of APCDD1, we silenced either of the transcription factors and noted that binding of the other was significantly impaired (Figure 6F, G; Supplementary Figure S11). We next cloned individual super-enhancer constituents (E2, E3, E4 and E5) of APCDD1 into the pGL3-Promoter luciferase reporter vector, and measured their activities by reporter assays. As shown in Supplementary Figure S12A and B, E2 and E4 were particularly active in both A673 and SKNMC cells (> 1.5-fold over control, *P* < 0.01), while E3 and E5 had little activities. Importantly, enhancer activities of both E2 and E4 were significantly decreased upon depletion of either MEIS1 or EWS-FLI1 in A673 and SKNMC cells (Supplementary Figure S12C). Moreover, both the mRNA and protein expressions of APCDD1 were substantially suppressed when silencing MEIS1 or EWS-FLI1 (Figure 7A, B). These findings suggested that MEIS1 and EWS-FLI1 may facilitate the recruitment of each other, and co-operatively activate the transcription of APCDD1 by its enhancer elements E2 and E4.

Finally, we tested whether super-enhancer-driven APCDD1 played a role in the biology of Ewing sarcoma. Similar to MEIS1 silencing, APCDD1 depletion reduced cell proliferation and anchorage-independent growth of Ewing sarcoma cells (Figure 7C, D). Moreover, APCDD1 depletion potently inhibited the growth of Ewing sarcoma xenografts (Supplementary Figures S6, 13). Considering the pro-growth functions observed in both MEIS1 and APCDD1, and the activation of APCDD1 transcription by MEIS1 and EWS-FLI1, we speculated that APCDD1 might function as a key factor mediating the oncogenic role of MEIS1. To address this, APCDD1 was ectopically expressed in MEIS1-silenced cells. Importantly, APCDD1 restoration mitigated significantly the growth-inhibitory effects triggered by MEIS1-silencing, although ectopic expression of APCDD1 did not increase MEIS1 expression level (Figure 7E). These results together identified APCDD1 as a prominent tumor-promoting factor associated with a super-enhancer in Ewing sarcoma. Moreover, our data suggested that MEIS1 and EWS-FLI1 co-operatively activate APCDD1 transcription, thereby promoting the malignant phenotype of Ewing sarcoma cells (Figure 7F).

## DISCUSSION

EWS-FLI1 is the central and driving force of tumorigenesis and progression of Ewing sarcoma (1). Unfortunately, clinical efficacy through directly targeting EWS-FLI1 has yet to be achieved (49). Endeavors aimed at identifying additional dependencies in Ewing sarcoma are expected to provide novel therapeutic avenues for treatment of this cancer. Here, we found that Ewing sarcoma cells were highly vulnerable to transcriptional perturbation achieved by CDK7 inhibition both *in vitro* and *in vivo* (Figure 1), which was independently confirmed by Iniguez *et al.*’s recent study (50). We also observed clusters of genes, which were enriched in key cellular functions associated with survival and proliferation of cancer cells, preferentially sensitive to THZ1 treatment in Ewing sarcoma cells (Figure 2C, D).

To investigate whether THZ1-sensitive transcripts were associated with any (epi)genomic features, the super-enhancer landscape was comprehensively characterized in Ewing sarcoma by analyzing published H3K27ac ChIP-seq data in both Ewing sarcoma cell lines and primary tumors (4,6). This approach highlighted the critical roles of super-enhancer-associated genes in conferring sensitivity of Ewing sarcoma cells to transcriptional inhibition (Figure 3B, C).

It has become increasingly clear that super-enhancers in cancer cells are often bound by master regulatory transcription factors which mediate oncogenic gene expression programs (6,51,52). In the context of Ewing sarcoma, we demonstrated that in comparison with typical-enhancers, super-enhancers had significantly more EWS-FLI1 binding peaks as well as its active motif, GGAA repeats (Figure 3D, E). Besides, super-enhancer-associated transcripts were enriched among genes whose expression exhibited the most profound changes following EWS-FLI1 depletion (Figure 3F). These results further supported the notion that super-enhancer transcripts are enriched in EWS-FLI1 target genes (6), and can be leveraged to identify novel oncogenes in Ewing sarcoma.

Our integrated analysis generated a total of 147 genes, which were associated with super-enhancers in both Ewing sarcoma cell lines and primary samples. Notably, on this list, we observed both well-established and candidate oncogenes in Ewing sarcoma, with many of which displayed striking Ewing sarcoma-specific expression profiles (e.g. LINGO1, HOOK1, CITED2 and IGF2BP1) (53,54) (Supplementary Figure S2C). Our results thus provided a category of potential oncogenic factors for future studies.

MEIS1 is a transcription factor belonging to the Three Amino Acid Loop Extension (TALE) family of homeodomain-containing proteins. Acting as a cofactor of homeobox (HOX) family members, MEIS1 has been extensively studied for its essential role in the differentiation of hematopoietic cells (35,45,55–57). However, whether and how MEIS1 functions in other tissue types is poorly explored. In acute leukemia, overexpression of MEIS1 has been consistently observed (36,39–43), and level of MEIS1 is inversely correlated with prognosis of this hematopoietic malignancy (58,59). In this study, we found that the expression level of MEIS1 in Ewing sarcoma cells was comparable with, or even higher than, that of leukemic cells (Supplementary Figure S3). Through a series of cellular phenotypical assays and in vivo experiment, we characterized MEIS1 in Ewing sarcoma as both a driver of cell proliferation and survival (Figure 5). Interestingly, recent publications revealed contributions of HOXD genes to the malignancy of Ewing sarcoma (60,61). Considering our observation from ChIP-seq experiment that the HOX motif was strongly enriched in MEIS1-interacting DNA sequence (Figure 6A), future exploration of the functional interplay between MEIS1 and HOX genes in the context of Ewing sarcoma will be of great interest.

Notably, another strongly enriched motif in MEIS1 ChIP-seq data was EWS-FLI1 (Figure 6A). Via analyzing the ChIP-seq results of MEIS1 and EWS-FLI1, as well as the RNA-seq results of EWS-FLI1 knockdown in A673 cells, we found approximately a quarter of MEIS1 peaks shared the same genomic binding regions with EWS-FLI1 (Figure 6B). Meanwhile, 13.8% of ‘EWS-FLI1 direct target genes’ were also co-occupied by MEIS1 (Supplementary Figures S7, S8 and Dataset S10). Thus, we speculated that in Ewing sarcoma, the disease-defining fusion protein EWS-FLI1 might act as a cell-type specific MEIS1-interacting factor, facilitating and co-operating with MEIS1 in regulating the gene expression program. In addition, we identified the super-enhancer-associated APCDD1, which was uniquely expressed at high levels in Ewing sarcoma, as a novel pro-growth gene transcriptionally activated by MEIS1 and EWS-FLI1 in a co-operative fashion. Specifically, MEIS1 and EWS-FLI1 both were recruited to super-enhancer elements of APCDD1, thereby promoting transcription of the latter (Figures 6C–G and 7; Supplementary Figure S12).

APCDD1 is a transmembrane glycoprotein that is associated with the Wnt/β-catenin signaling pathway. However, the functional regulation of APCDD1 on Wnt/β-catenin pathway is considerably cell type- and tissue context-dependent (34,62,63). In this study, we showed that APCDD1 knockdown increased the expression of CDC25A, LRP6, LEF1 and NKD1, which are confirmed targets of the canonical Wnt pathway in Ewing sarcoma (64–68) (Supplementary Figure S14), implying that APCDD1 might act as a Wnt inhibitor in this cancer. Nevertheless, future investigations are needed to dissect its functional mechanisms in Ewing sarcoma.

In summary, the current study provides comprehensive analysis of super-enhancer-mediated transcriptional dysregulation of Ewing sarcoma, and identifies MEIS1 as a novel super-enhancer-driven oncogene, which co-operates with EWS-FLI1 in transcriptional regulation. Importantly, our data suggest that targeting the super-enhancer-associated oncogenic transcription program may serve as a promising therapeutic method for Ewing sarcoma, and highlight super-enhancer-associated genes as a valuable resource for future research of the pathogenesis of Ewing sarcoma.

## DATA AVAILABILITY

The MEIS1 ChIP-seq raw data generated in A673 Ewing sarcoma cell line is available on NCBI GEO under the accession number ‘GSE109477’; the RNA-seq raw data of A673 and SKNMC cells upon DMSO or THZ1 treatment are also available on GEO, under the accession number ‘GSE117485’.

## Supplementary Material

Supplementary Data
