# Supplementary material for: Super-enhancer-associated MEIS1 promotes transcriptional dysregulation in Ewing sarcoma in co-operation with EWS-FLI1
Source: Nucleic Acids Res. 2018 Nov 28;47(3):1255–67. doi: 10.1093/nar/gky1207 (PMC6379679; doi:10.1093/nar/gky1207)
Supplement: Supplementary Data [file gky1207_supplemental_files.zip › Supplementary Data.pdf]

Supplemental Materials and Methods

MTT cell proliferation assay

Ewing sarcoma cells were seeded into 96-well plates at a density of 3,000 to 4,000 cells per well and cultured for indicated time course before MTT (3-(4, 5-dimethylthiazol-2-yl)-2, 5-diphenyltetrazolium bromide) assay was performed to detect cell viability. Specifically, for THZ1 treatment, the cultural medium was replaced with fresh complete medium containing either DMSO or THZ1 at day 0 once the cells were attached to the plates. For gene functional evaluation, cells containing indicated siRNAs or shRNAs were seeded at day 0, and assayed at day 1, 3 and 5. To measure cell proliferation, 20 μl of MTT substrate (Sigma Aldrich) was added into each well and incubated for 4 hours followed by addition of 100 μl of MTT STOP solution (2% acetic acid, 16% SDS, 42% dimethylformamide). Plates were read with the absorbance at 570 nm using a SpectraMax M5 (Molecular Devices).

Apoptosis assay

For detection of apoptotic cells, the FITC Annexin V Apoptosis Detection Kit (BD Biosciences) was used according to the manufacturer's instructions. Briefly, cells were harvested and washed twice with cold PBS, resuspended in 1X Annexin V Binding Buffer to reach a concentration of 1×10^6 cells/ml. 100 μl of the solution (1×10^5 cells) were then transferred and added with 5 μl of FITC Annexin V and 5 μl propidium iodide (PI), and incubated for 15 min at room temperature in the dark. Finally, 400 μl of 1X Annexin V Binding Buffer were added and cells were analyzed

using a BD FACSCanto II flow cytometer. Data were analyzed using FlowJo 7.6 software (Tree Star).

Xenograft assay

All animal experiments were conducted following protocols approved by the Institutional Animal Care and Use Committee (IACUC) of Cedars-Sinai Medical Center.

For efficacy study of THZ1 treatment in A673 tumor xenografts, twelve seven-week-old BALB/c-nu female mice (Taconic Bioscience) were subcutaneously inoculated in their dorsal flanks with a suspension of A673 cells (2.0×10^6) in matrigel. One week after injection when tumor xenografts were palpable, mice were randomized into two groups, and treated with either vehicle or THZ1 (10 mg/kg, twice daily) by intraperitoneal injection (IP).

For efficacy study of MEIS1 or APCDD1 knockdown in A673 xenografts, A673 cells were engineered to stably express doxycycline (DOX)-inducible shRNAs (A673-Tet-shMEIS1 and A673-Tet-shAPCDD1), and were cultured in DMEM supplemented with 10% Tetracycline (Tet)-free FBS (Biological Industries) before implantation. Xenograft tumors were established in seven-week-old BALB/c-nu female mice (Taconic Bioscience) by subcutaneously inoculated in their dorsal flanks with a suspension of A673-Tet-shMEIS1 or A673-Tet-shAPCDD1 cells (2.0×10^6) in matrigel. After implantation, mice were randomized into two groups (n=5), and received either vehicle (3% sucrose in drinking water, ad libitum) or doxycycline hyclate (25 mg/kg per day via oral gavage) for the duration of the experiments.

General behaviors of all experimental mice were monitored every day; and tumor volumes and body weights were measured every 4 days. Tumor volumes were calculated using the formula: $V\ (mm^3) = [width\ (mm)]^2 \times length\ (mm)/2$, as described previously (1,2). Mice were euthanized by CO_2 inhalation when the largest tumors were approximately 1.5 cm in diameter; tumor xenografts were surgically dissected and weighed.

Chromatin immunoprecipitation (ChIP) assay

ChIP was performed by following a standard protocol. Briefly, 2×10^7 of A673 cells were crosslinked with 1% formaldehyde for 10 min at room temperature and quenched by glycine (final concentration: 125 mM). Cells were washed 3 times by cold PBS, collected by spinning down at 2,000 rpm for 5 min, and re-suspended in nuclear extraction buffer to enrich nuclei. Nuclei were subjected to SDS lysis buffer for 10 min on ice and sheared in a Bioruptor Sonicator (Diagenode) to achieve genomic fragments around 200 to 500 base pairs. After sonication, cell debris was removed by centrifugation at 13,000 rpm for 10 min. Supernatant was then incubated with 5 μg of anti-MEIS1 antibody, anti-FLI-1 antibody, or IgG control overnight at 4℃ on a rotating wheel. Antibody-chromatin complexes were subsequently pulled down by further incubation with Dynabeads Protein G (Life Technologies) for four hours at 4℃, and followed with increasing stringent washes with cold low salt wash buffer, high salt wash buffer, LiCl wash buffer and TE buffer. Finally, bound DNA was eluted by elution buffer (1% SDS, 100 mM NaHCO3), reverse-crosslinked overnight at 65℃, treated with RNase A and Proteinase K, purified by the Min-Elute PCR

purification kit (Qiagen), and subjected to qPCR analysis or sequencing on Illumina HiSeq 3000 platform. Primers for qPCR analysis are listed in Supplementary Table S2.

qRT-PCR

Total RNA was extracted from cells using RNeasy mini kit (Qiagen) following the manufacturer's instructions, and 1 μg aliquots were used for cDNA synthesis with the qScript™ cDNA Synthesis Kit (Quanta Biosciences), yielding 20 μl of cDNA solutions. The cDNA products were diluted 10-fold with 10 mM Tris-HCl (pH 8.0) and 0.1 mM EDTA for subsequent qRT-PCR analysis. PCR amplification was conducted with 1 μl of RT product in a 20 μl reaction on CFX96 qPCR System (Biorad). PCR Conditions were as follows: 95℃ for 3 min, followed by 36 cycles at 95℃ for 15 s, 60℃ for 30 s, and 68℃ for 30s, with a final extension at 68℃ for 5 min. After the cycling protocol, melting curves were generated by continuously monitoring fluorescence through the dissociation temperature of the PCR product at a temperature transition rate of 0.1℃/s. Expression of each gene was normalized to GAPDH, and quantified using the $2^{-delta(ct)}$ method. Primers are listed in Supplementary Table S3.

RNA-seq and data analysis

RNA-seq was performed as described (3,4). Briefly, sequencing libraries were prepared using TruSeq Library Prep Kit (Illumina) according to the manufacturer's protocol, and were sequenced on HiSeq2000 sequencer (Illumina). 100-bp paired-end reads were aligned to hg19 reference genome with Ensemble gtf (version 75)

provided as a known junction file, using splice aware STAR aligner. Expression level was measured as FPKM using stringtie software against ensemble v75 gtf.

ChIP-sequencing (ChIP-seq) data analysis

Raw sequencing reads passing quality control were aligned to human reference genome (hg19/ GRCh37) using Bowtie Aligner (version 1.1.2). PCR duplicates were marked with picard MarkDuplicates and removed from further analysis. ChIP-seq peaks were called using MACs (Model-Based Analysis of ChIP-seq, version 2.1.0) with default parameter setting. Wiggle files were generated and normalized at the unit of reads per million reads (rpm). These files were converted into bigwig files using wigToBigWig tool (http://hgdownload.cse.ucsc.edu/admin/exe/) and visualized in Integrative Genomics Viewer (http://www.broadinstitute.org/igv/home).

H3K27ac ChIP-seq data generated in 4 Ewing sarcoma cell lines and 3 primary tumors were retrieved from NCBI Gene Expression Omnibus (GEO) or generously shared by Dr. Kimberly Stegmaier (Harvard Medical School) and processed uniformly. MACs peaks of H3K27ac were used as constituent enhancers for super-enhancer identification by the ROSE (Rank Ordering of Super-enhancers, https://bitbucket.org/young_computation/rose) tool as previously described (5,6). Briefly, closely spaced enhancers (except those within +/- 2 kb of any Transcription Start Site) within 12.5 kb of each other were stitched together. All enhancers were then ranked in increasing order of input-subtracted H3K27ac signal, and were geometrically classified into super-enhancers or typical-enhancers by an inflection

point (tangent slope = 1). Both super-enhancers and typical-enhancers were assigned to the nearest ensemble genes.

Gene Set Enrichment Analysis (GSEA)

GSEA for super-enhancer- and typical-enhancer-associated transcripts of Ewing sarcoma was performed as previously described (7,8). Briefly, GSEA standalone desktop programme was used. An expression matrix was created containing gene expression values at 0 h and 6 h upon 50 nM THZ1 treatment (Fig. 3B), or gene expression values in either the presence or absence of shEWS-FLI1 (Fig. 3F). In both cases, all super-enhancer-associated genes were used as a "gene set database". GSEA was run with parameter "Metric for ranking genes" set to "log2_Ratio_of_classes" to calculate enrichment score for super-enhancer-associated genes.

Statistical analysis

Data were presented as mean ± SD of three independent experiments. Two-tailed Student's t-test and one-way analysis of variance were used to evaluate the data. All statistical analyses were performed with SPSS 19.0. Differences were considered statistically significant at $p<0.05$ (*), $p<0.01$ (**) and $p<0.001$ (***). Diagrams were created by GraphPad Prism 6 software.

Data availability

The MEIS1 ChIP-seq raw data generated in A673 cells, and RNA-seq raw data of A673 and SKNMC cells upon DMSO or THZ1 treatment have been deposited into the GEO under accession numbers "GSE109477" and "GSE117485", respectively.

The H3K27ac ChIP-seq raw data for TC32 and TC71 cells were generously shared by Dr. Kimberly Stegmaier (Harvard Medical School). All the other raw data used in this study, including ChIP-seq of EWS-FLI1, H3K27ac, H3K4me1 and H3K4me3 in A673 and SKNMC cells, ChIP-seq of H3K27ac in three Ewing sarcoma primary tumors, and RNA-seq for A673 and SKNMC cells in either the presence or absence of EWS-FLI1 knockdown were obtained from GEO under accession number "GSE61953".

References

1. Sun, H., Lin, D.C., Cao, Q., Guo, X., Marijon, H., Zhao, Z., Gery, S., Xu, L., Yang, H., Pang, B. *et al.* (2016) CRM1 Inhibition Promotes Cytotoxicity in Ewing Sarcoma Cells by Repressing EWS-FLI1-Dependent IGF-1 Signaling. *Cancer research*, **76**, 2687-2697.
2. Sun, H., Lin, D.C., Cao, Q., Pang, B., Gae, D.D., Lee, V.K.M., Lim, H.J., Doan, N., Said, J.W., Gery, S. *et al.* (2017) Identification of a Novel SYK/c-MYC/MALAT1 Signaling Pathway and Its Potential Therapeutic Value in Ewing Sarcoma. *Clinical cancer research : an official journal of the American Association for Cancer Research*, **23**, 4376-4387.
3. Lin, D.C., Hao, J.J., Nagata, Y., Xu, L., Shang, L., Meng, X., Sato, Y., Okuno, Y., Varela, A.M., Ding, L.W. *et al.* (2014) Genomic and molecular characterization of esophageal squamous cell carcinoma. *Nature genetics*, **46**, 467-473.
4. Yuan, J., Jiang, Y.Y., Mayakonda, A., Huang, M., Ding, L.W., Lin, H., Yu, F., Lu, Y., Loh, T.K.S., Chow, M. *et al.* (2017) Super-enhancers promote transcriptional dysregulation in nasopharyngeal carcinoma. *Cancer research*.
5. Loven, J., Hoke, H.A., Lin, C.Y., Lau, A., Orlando, D.A., Vakoc, C.R., Bradner, J.E., Lee, T.I. and Young, R.A. (2013) Selective inhibition of tumor oncogenes by disruption of super-enhancers. *Cell*, **153**, 320-334.
6. Hnisz, D., Abraham, B.J., Lee, T.I., Lau, A., Saint-Andre, V., Sigova, A.A., Hoke, H.A. and Young, R.A. (2013) Super-enhancers in the control of cell identity and disease. *Cell*, **155**, 934-947.
7. Jiang, Y.Y., Lin, D.C., Mayakonda, A., Hazawa, M., Ding, L.W., Chien, W.W., Xu, L., Chen, Y., Xiao, J.F., Senapedis, W. *et al.* (2017) Targeting super-enhancer-associated oncogenes in oesophageal squamous cell carcinoma. *Gut*, **66**, 1358-1368.

8. Subramanian, A., Tamayo, P., Mootha, V.K., Mukherjee, S., Ebert, B.L., Gillette, M.A., Paulovich, A., Pomeroy, S.L., Golub, T.R., Lander, E.S. *et al.* (2005) Gene set enrichment analysis: a knowledge-based approach for interpreting genome-wide expression profiles. *Proceedings of the National Academy of Sciences of the United States of America*, **102**, 15545-15550.

Supplementary Figure legends

Figure S1. Super-enhancer profiling in TC32 and TC71 Ewing sarcoma cell lines and three primary tumors

Hockey stick plots showing rank order of H3K27ac signals for all enhancers in (A) TC32 and TC71 Ewing sarcoma cell lines as well as (B) three primary tumors. Inserted panels showing selected GO functional categories of super-enhancer-associated genes.

Figure S2. Nomination of super-enhancer-associated transcripts in Ewing sarcoma

(A-B) Tracks showing ChIP-seq signals of EWS-FLI1, H3K27ac, H3K4me1 and H3K4me3 at representative super-enhancer-associated gene loci across Ewing sarcoma cell lines and primary tumors. Y axis represents the value of reads per million per base pair (rpm/bp). (C) Data retrieved from CCLE project depicting mRNA expression of representative super-enhancer-associated transcripts across various types of human cancer cells.

Figure S3. Immunoblotting comparing the protein expression levels of MEIS1 in cells from Ewing sarcoma, breast cancer and leukemia.

Figure S4. Luciferase reporter activity of enhancer constituents of MEIS1 measured in A673 and SKNMC cells.

(A) H3K27ac ChIP-seq binding profiles for MEIS1 in A673 and SKNMC cells. Genomic locations of NC and E1-7 were shown (NC, Negative Control). (B) A673 and SKNMC cells were transfected with a luciferase reporter gene driven by any of the following constituents: NC, E1, E2, E3, E4, E5, E6 and E7. Bars represent normalized luciferase reporter activity, which was calculated through dividing the luciferase activity by the Renilla activity. Error bars represent mean ± SD of three replicates (NC, Negative Control).

Figure S5. Immunoblotting showing protein levels of MEIS1 were decreased by THZ1 treatment (50 nM) in A673 and SKNMC cells.

Figure S6. A673 cells stably transduced with either inducible scramble shRNA (Tet-Scramble) or MEIS1/APCDD1 shRNAs (Tet-shMEIS1 or Tet-shAPCDD1) were cultured in either the presence or absence of doxycycline (DOX) at 100 ng/ml for 72 h. Cells were then harvested for (A) qRT-PCR or (B) immunoblotting analysis (n.s., statistically no significance, ***$P<0.001$).

Figure S7. Venn diagram of the identification of 513 EWS-FLI1 direct target genes. "RNA-seq DE genes" refers to genes with expression change >2-fold upon EWS-FLI1 knockdown (DE, differential expression).

Figure S8. Pie chart depicting 71 EWS-FLI1 direct target genes which were also co-bound by MEIS1.

Figure S9. qRT-PCR measuring mRNA levels of indicated genes upon knockdown of either MEIS1 or EWS-FLI1 in A673 and SKNMC cells. Error bars represent mean ± SD of three replicates.

Figure S10. Integrative Genomics Viewer showing the ChIP-seq profiles of MEIS1, EWS-FLI1, H3K27ac, H3K4me1 and H3K4me3 at STEAP1 locus in A673 and SKNMC cells.

Figure S11. Immunoblotting showing the knockdown efficiency of (A) EWS-FLI1 and (B) MEIS1 in A673 and SKNMC cells (corresponding to Figures 6F, G).

Figure R12. (A) ChIP-seq binding profiles for MEIS1 and EWS-FLI1 at APCDD1 locus in A673 cells. (B) A673 and SKNMC cells were transfected with a luciferase reporter gene driven by any of the following constituents: NC, E2, E3, E4 and E5. Bars represent normalized luciferase reporter activity, which was calculated through dividing the luciferase activity by the Renilla activity. Error bars represent mean ± SD

of three replicates (NC, Negative Control). (C) Luciferase activities of E2 and E4 upon knockdown of either MEIS1 or EWS-FLI1 in A673 and SKNMC cells. Error bars represent mean ± SD of three replicates (** P<0.01, *** P<0.001, NC, Negative Control).

Figure S13. Nude mice were inoculated subcutaneously with A673 cells stably expressing inducible APCDD1 shRNA (A673-Tet-shAPCDD1). Mice were randomly allocated to either doxycycline (DOX)-treated or vehicle-treated groups (n=5 per group). (A) Representative images and (B) weights of resected tumors at the end point (*P<0.05).

Figure S14. qRT-PCR measuring the expression of established canonical Wnt/β-catenin target genes in APCDD1-silenced A673 and SKNMC cells. Error bars represent mean ± SD of three replicates (** P<0.01, *** P<0.001).

Supplementary Figure S1

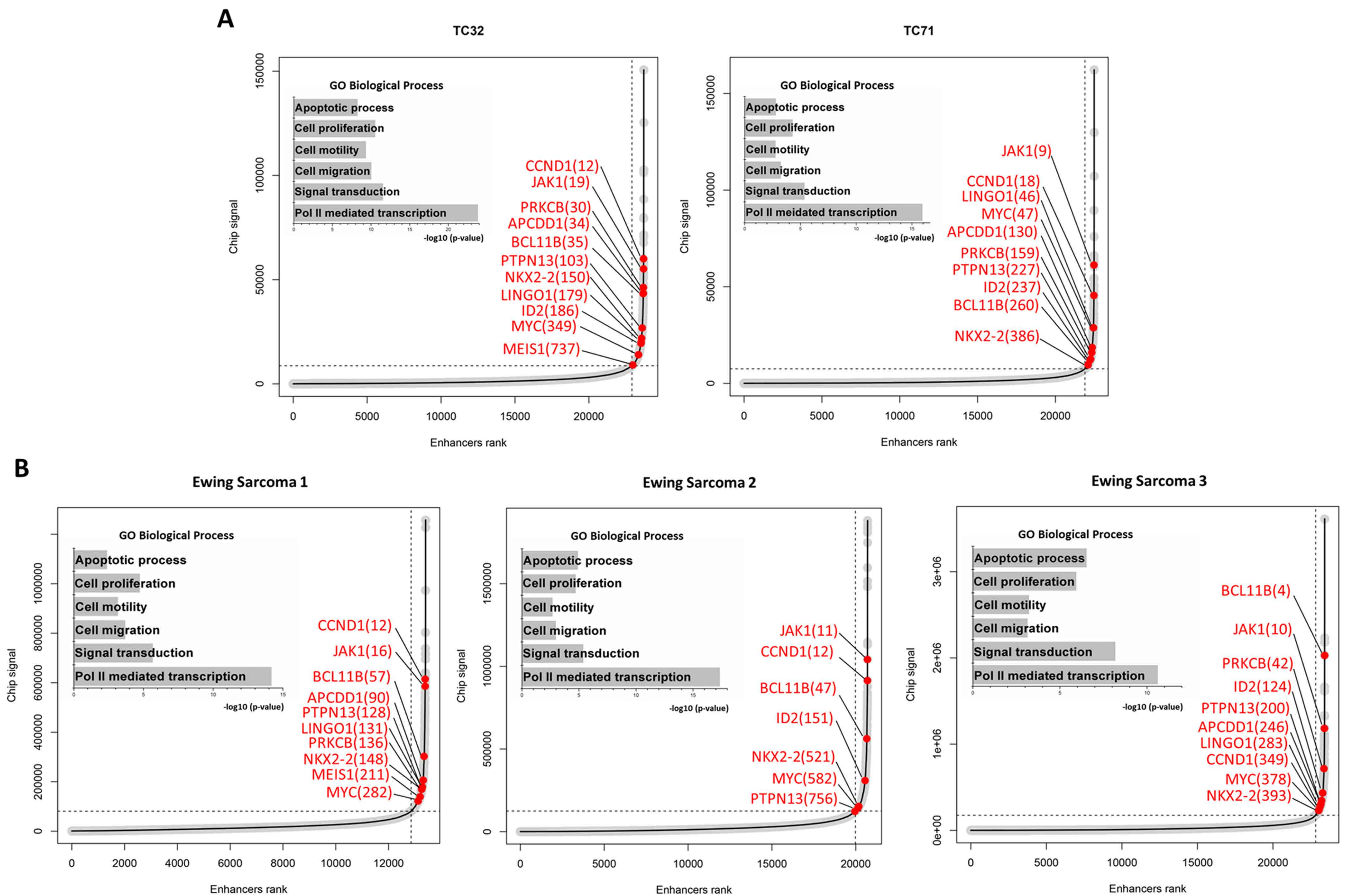

Supplementary Figure S2

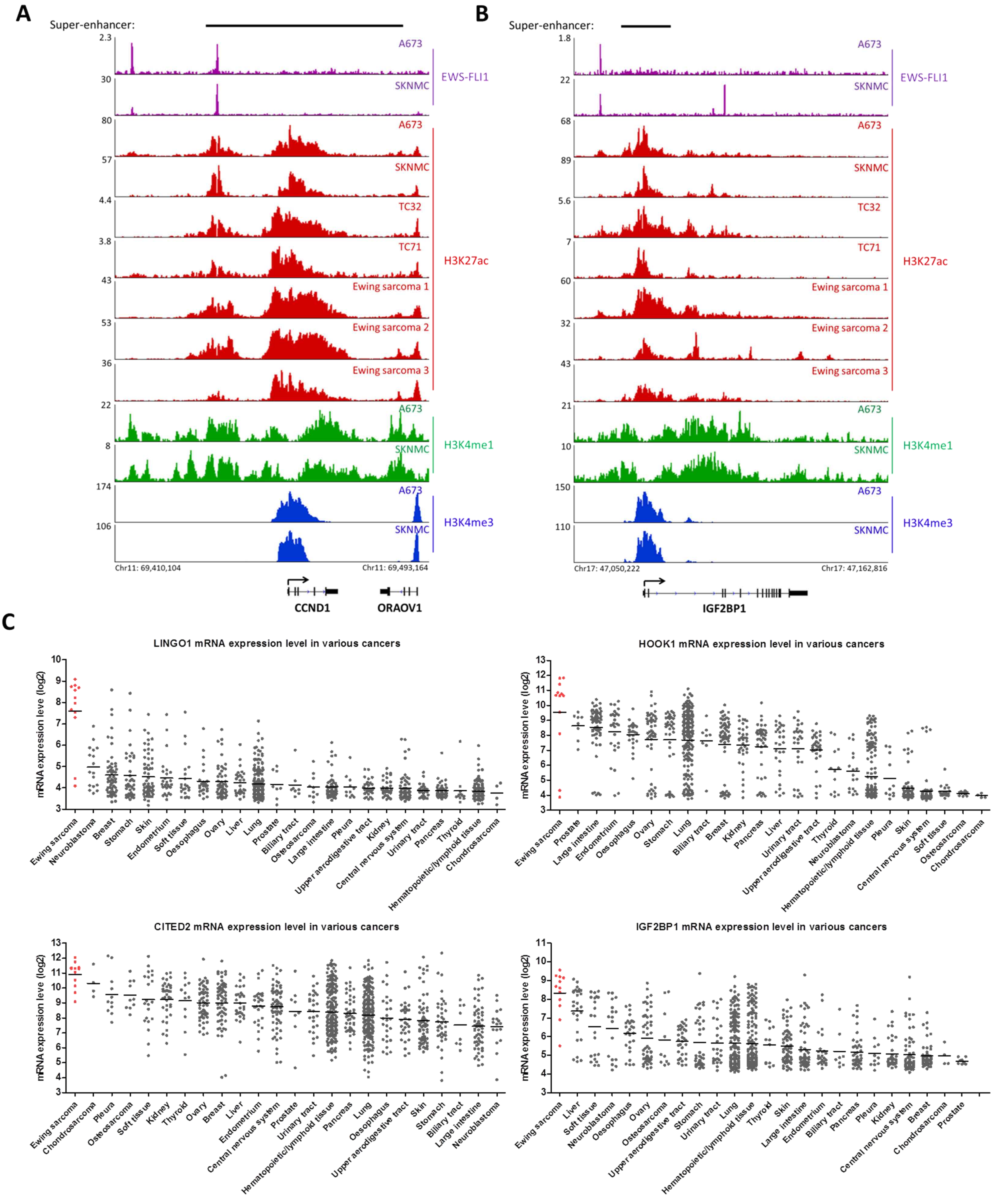

Supplementary Figure S3

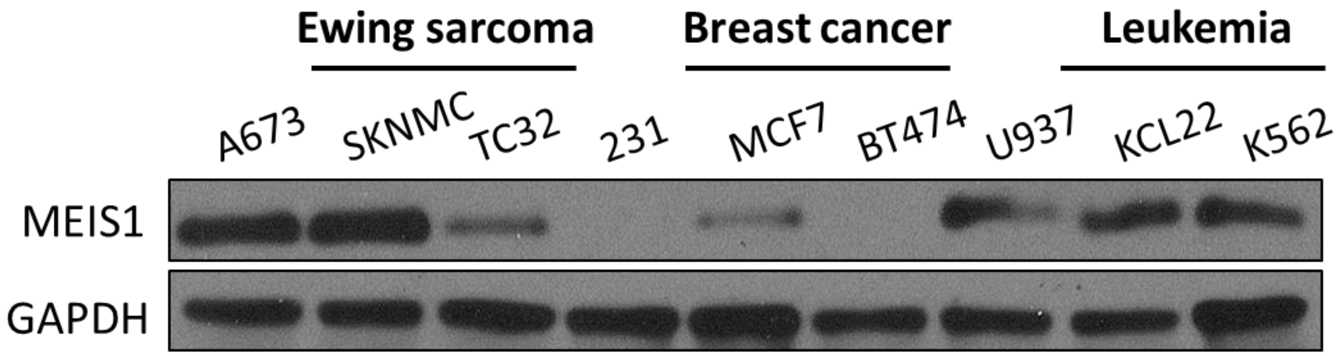

Supplementary Figure S4

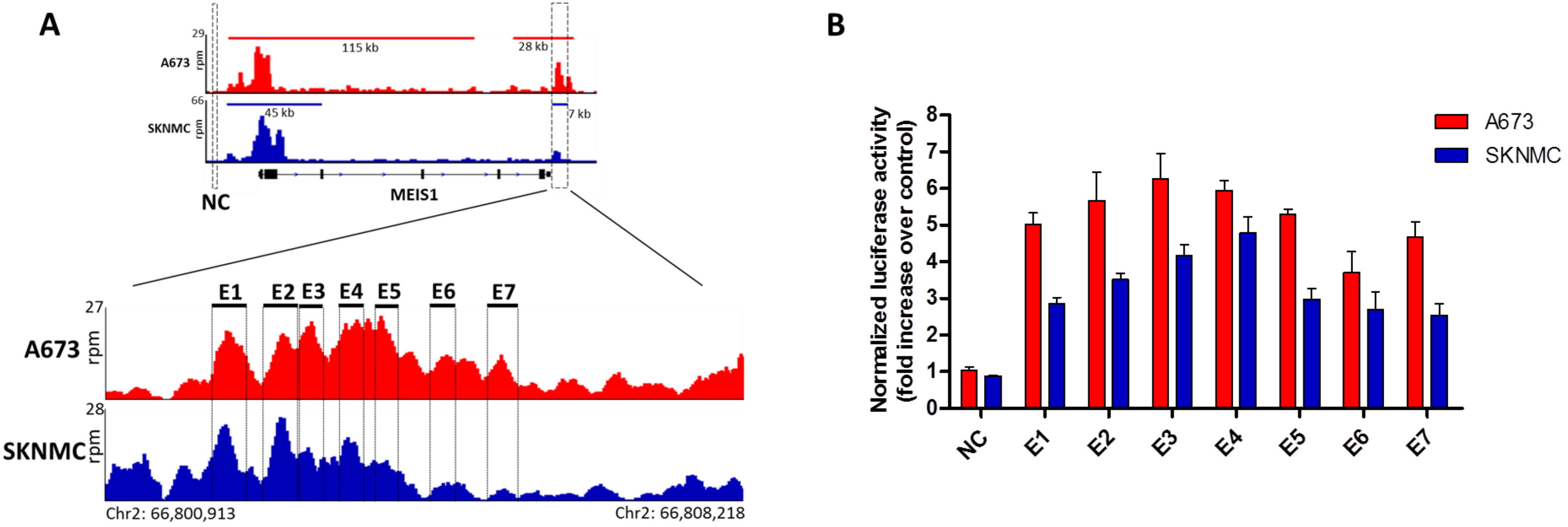

Supplementary Figure S5

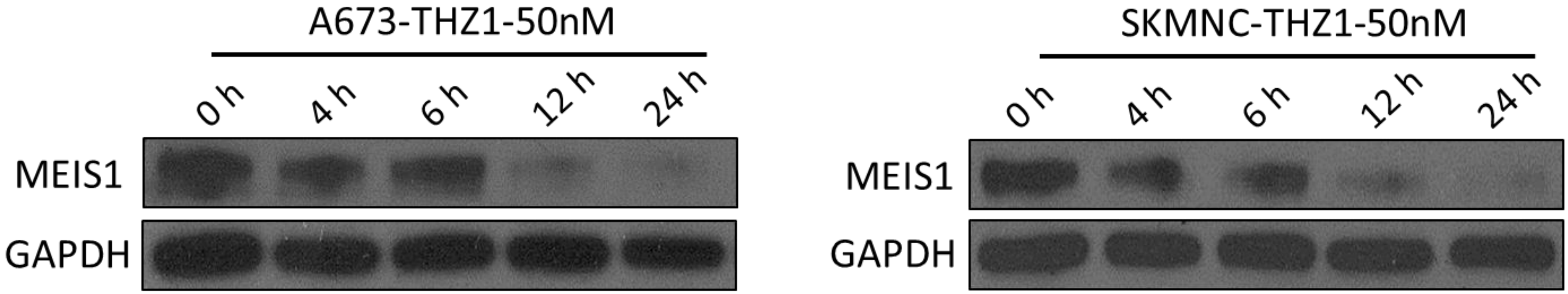

Supplementary Figure S6

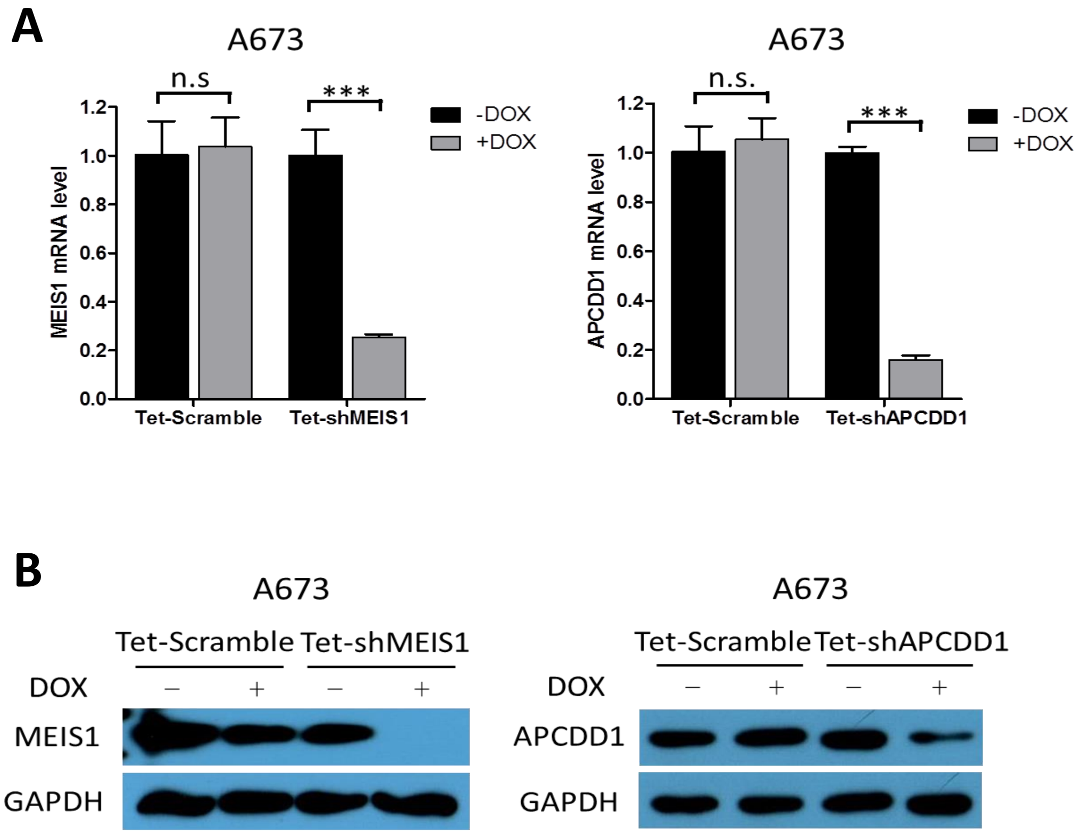

Supplementary Figure S7

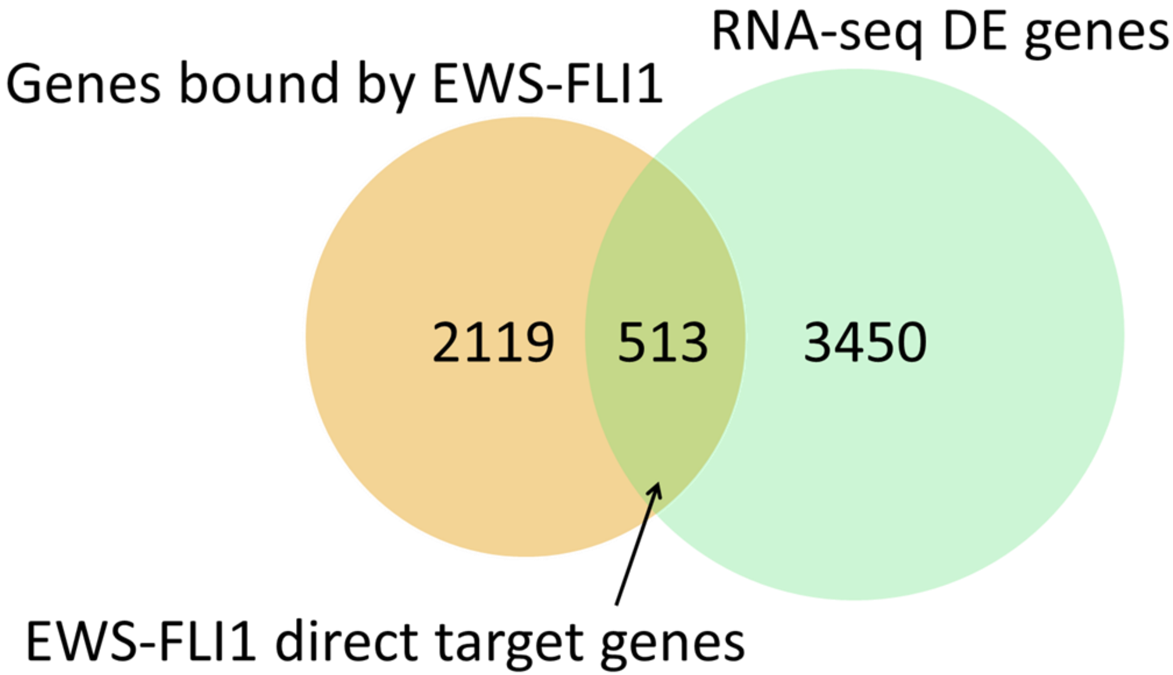

Supplementary Figure S8

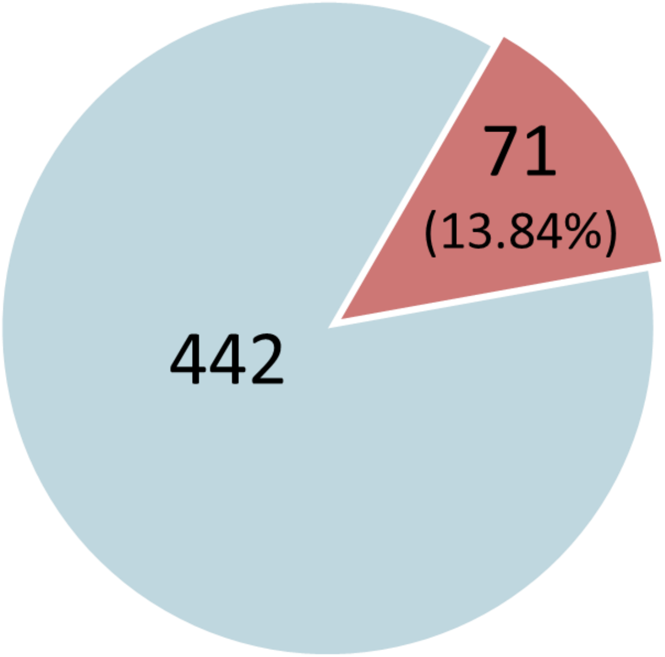

Supplementary Figure S9

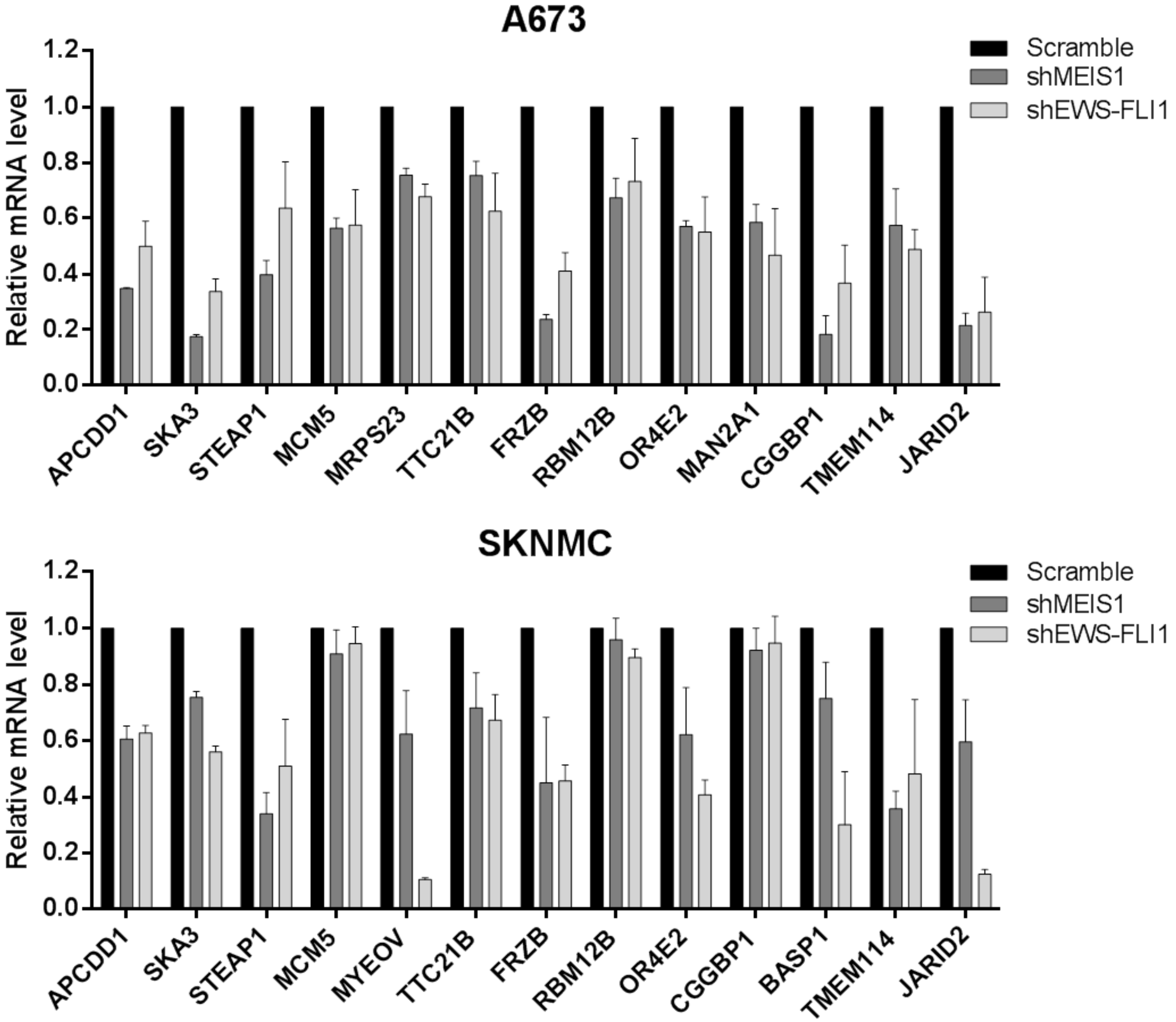

Supplementary Figure S10

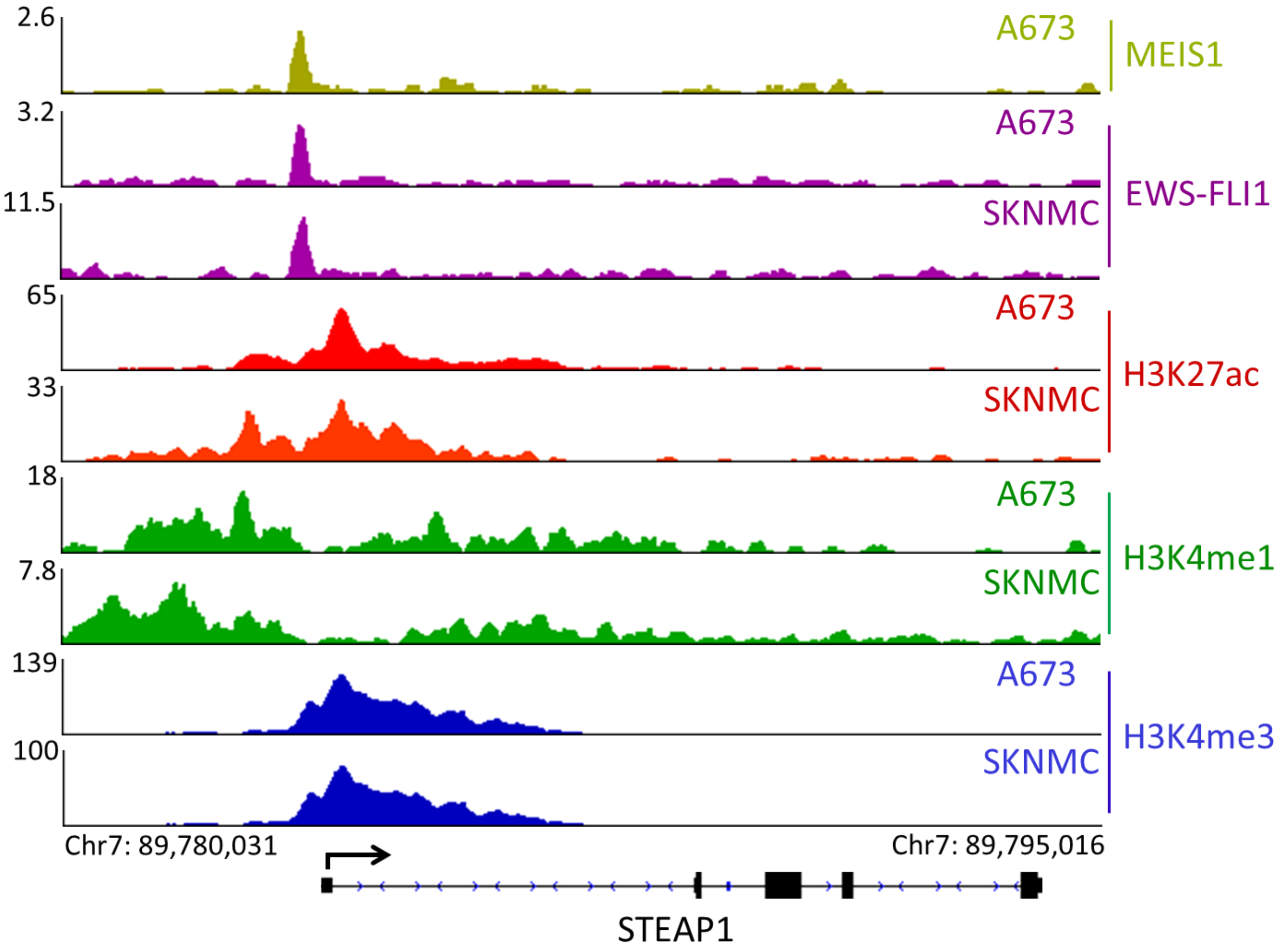

Supplementary Figure S11

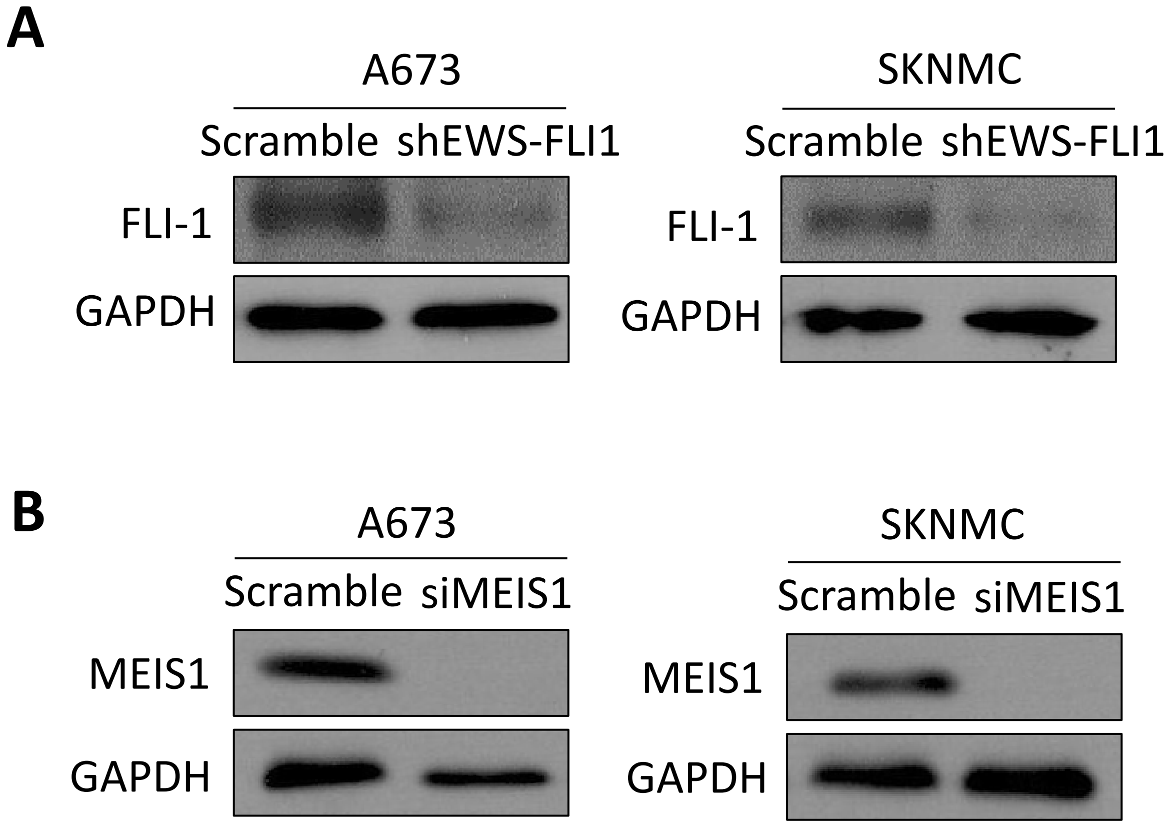

Supplementary Figure S12

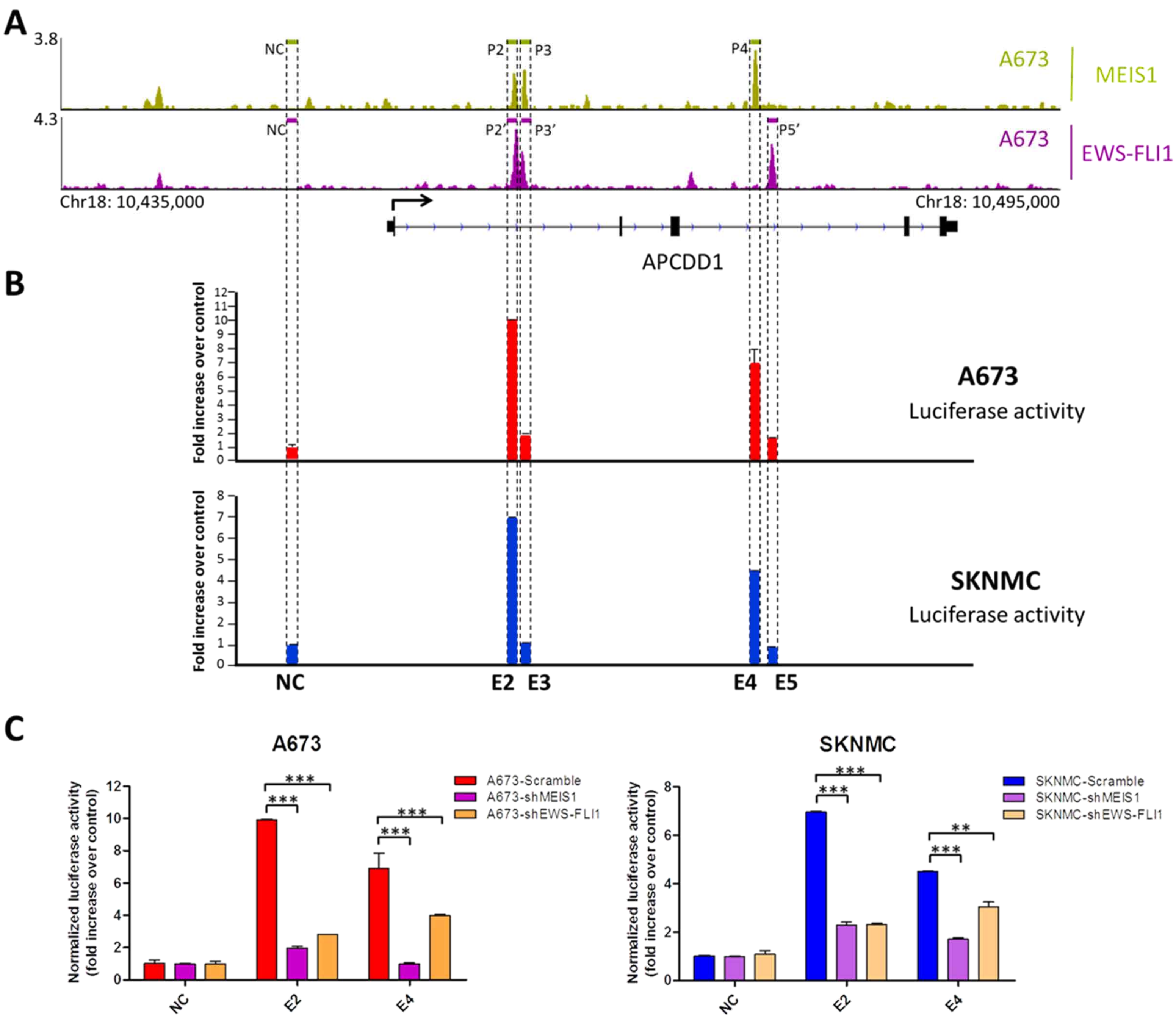

Supplementary Figure S13

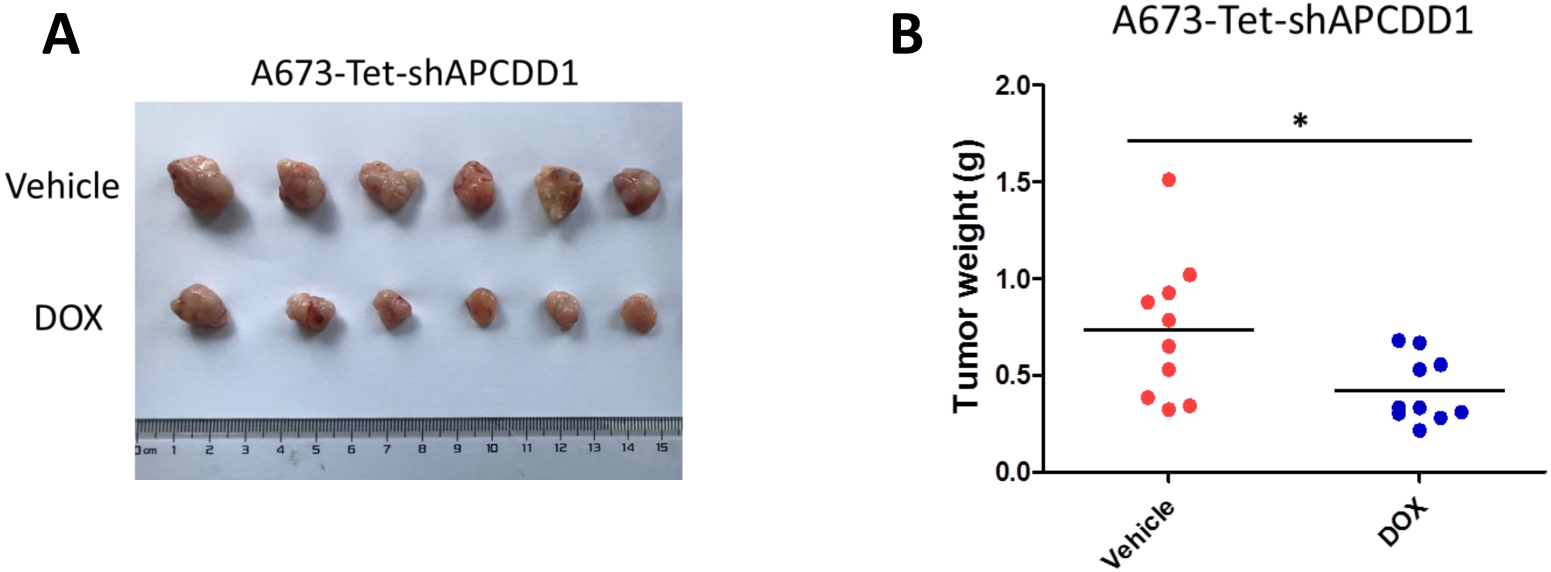

Supplementary Figure S14

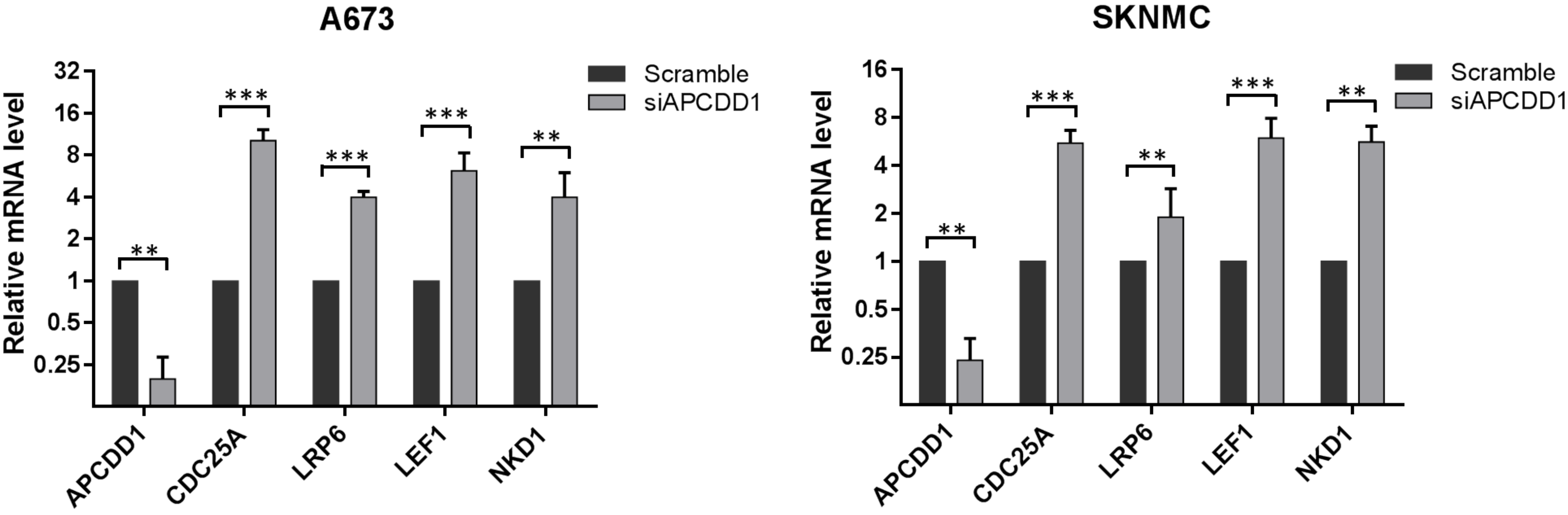

Supplementary Table S1

List of siRNA target sequences

Target Gene	siRNA target sequences (5’→3’)
MEIS1	CAAUGACGCUUUAAAGAGA
	GAUAGACGAUAGAGAAGGA
	GCAAAGGUACGACGAUCUA
	UAGAGAAGGUACACGAAUU
APCDD1	GCUCAAACAUCUCCACAAU
	CUUCAAGGCCUACCAAUUU
	CAAACUACCUCACACGGAG
	GCCCAGAGUUCAUCACAAG
siGENOME Non-Targeting siRNA Pool	UAAGGCUAUGAAGAGAUAC
	AUGUAUUGGCCUGUAUUAG
	AUGAACGUGAAUUGCUCAA
	UGGUUUACAUGUCGACUAA

Supplementary Table S2

List of ChIP-qPCR primers

Primers	Sequence (5’→3’)
APCDD1-P1-F	CACTTCTGAGGCTGTGAGGA
APCDD1-P1-R	CCCTAGAGGCCAATGATAGACT
APCDD1-P2-F	TGTACTCACACGGGAATGGT
APCDD1-P2-R	AGACAGGAAGGAAGGAAGGA
APCDD1-P3-F	GGTCATTGCCTCTCCAACAC
APCDD1-P3-R	GGGGACAGATTGAAAGCAGG
APCDD1-P4-F	GCTCATTCTTTCAGGGATGGA
APCDD1-P4-R	GGGAAGGCTGTCTGGTTTTC
APCDD1-P1’-F	CACTTCTGAGGCTGTGAGGA
APCDD1-P1’-R	CCCTAGAGGCCAATGATAGACT
APCDD1-P2’-F	TGTACTCACACGGGAATGGT
APCDD1-P2’-R	AGACAGGAAGGAAGGAAGGA
APCDD1-P3’-F	TCCTTCCTTCCCTCCTTCCT
APCDD1-P3’-R	GTGTTGGAGAGGCAATGACC
APCDD1-P4’-F	GGACTAAGGAGAGCAGGAAACA
APCDD1-P4’-R	GGGAGATGGTGGAGGTGATC
APCDD1-P5’-F	TCAGGGACAATGCAAGTGGT
APCDD1-P5’-R	GGCCCATCCTCTTCCTTTCT
APCDD1-NC-F	CTTCCAGTGCCACTTTTCCC
APCDD1-NC-R	CGCCATAAAACCTGCCTTGT

Supplementary Table S3

List of qRT-PCR primers

Primers	Sequence (5'→3')
GAPDH-F	GTCAGTGGTGGACCTGACCT
GAPDH-R	AGGGGTCTACATGGCAACTG
APCDD1-F	AATGCCAAGAACCACGACCA
APCDD1-R	GAAGATGAAGTGGCGGGTGA
MEIS1-F	GCGCAAAGGTACGACGATCT
MEIS1-R	GGTACTGATGCGAGTGCAGA
EWS-FLI1-F	GAGGCCAGAATTCATGTTATTGC
EWS-FLI1-R	GCCAAGCTCCAAGTCAATATAGC
SKA3-F	GCTCAGCATGGACCCTATCC
SKA3-R	TCAAAGTCGCTTTCCTCTCCG
STEAP1-F	TGGGCATATCAACAGGTCCAA
STEAP1-R	AATGCGTGTATTGTGCCCAG
MCM5-F	TCACCAAGCAGAAATACCCG
MCM5-R	CGACTCACTTGAGGCGGTAG
MRPS23-F	ACCTGTCAACGGTTTGTGGA
MRPS23-R	CCTCCAAATGCTGGTCCTGT
TCC21B-F	AGCTGAATTCTGCGTCTTCCT
TCC21B-R	TCAAAGTCTTCAATTCCTGCGAG
FRZB-F	CGGACGGAGCTGATTTTCCT
FRZB-R	GGCAGCCAGAGCTGGTATAG
RBM12B-F	TGGGCTCACTGAGGGTCTAC
RBM12B-R	TGTAAAAGCAACTTAACACTGCTG
OR4E2-F	ATGCTAACGCTTTCGGGGAA

OR4E2-R	AAGATCTCGGCACAGGCAAA
MAN2A1-F	TGTTTACACAACACCGCCCT
MAN2A1-R	CAGACTGTCCTTCTATTCCCTGT
CGGBP1-F	GGCCTCGATCCTACGTAAAGG
CGGBP1-R	CAGACAGTCATGGATCCAGAAGA
TMEM114-F	TATCATTGACACCGAGCGGC
TMEM114-R	CATGCATGGTGAGAAGTTGCC
JARID2-F	TTGCTTCGTTCGTCTTTGGC
JARID2-R	TCCCATCACTGTCATCGTATTTCT
MYEOV-F	AGATGCCATCCCCACTGAAC
MYEOV-R	GCTCCTGAATGGACTCTGCTC
BASP1-F	CAGGAAGGGGAGGGGGAG
BASP2-R	CCCATCTTGGAGTTCGGCTC
LRP6-F	CTGAGAGCGGCCCCTTTGTT
LRP6-R	GCCCATCGGGGGACAATAAT
LEF1-F	CTGGCAAGGTCAGCCTGTAT
LEF1-R	AGGCTTCACGTGCATTAGGT
CDC25A-F	CCCTACCTAAGCTGTTGGGATGTA
CDC25A-R	CAGAGTTCTGCCTCTGTGTGA
NKD1-F	GGCTGGAAGTGGCCCTG
NKD1-R	GCAAGCTGGTGATGTCCTCT

Supplementary Table S4

List of Oligos used for shRNA cloning

Gene	Oligo Name	Oligo Sequence (5’→3’)
CDK7	shCDK7-1F	CCGGCAACCAAATTGTCGCCATTAACTCGAGTTAATGGCGACAATTTGGTTGTTTTTG
	shCDK7-1R	AATTCAAAAACAACCAAATTGTCGCCATTAACTCGAGTTAATGGCGACAATTTGGTTG
	shCDK7-2F	CCGGTAATCCATGTGCTCGAATTACCTCGAGGTAATTCGAGCACATGGATTATTTTTG
	ShCDK7-2R	AATTCAAAAATAATCCATGTGCTCGAATTACCTCGAGGTAATTCGAGCACATGGATTA
MEIS1	shMEIS1-1F	CCGGGCCGTGTGTTTAGAAGCCTAACTCGAGTTAGGCTTCTAAACACACGGCTTTTTG
	shMEIS1-1R	AATTCAAAAAGCCGTGTGTTTAGAAGCCTAACTCGAGTTAGGCTTCTAAACACACGGC
	shMEIS1-2F	CCGGCCCTCTTGGAACAGAGATCATCTCGAGATGATCTCTGTTCCAAGAGGGTTTTTG
	shMEIS1-2R	AATTCAAAAACCCTCTTGGAACAGAGATCATCTCGAGATGATCTCTGTTCCAAGAGGG
EWS-FLI1	shEWS-FLI1-F	CCGGCGTCATGTTCTGGTTTGAGATCTCGAGATCTCAAACCAGAACATGACGTTTTTG
	shEWS-FL1-R	AATTCAAAAACGTCATGTTCTGGTTTGAGATCTCGAGATCTCAAACCAGAACATGACG
MEIS1	Tet-shMEIS1-F	CCGGCAATGACGCTTTAAAGAGACTCGAGTCTCTTTAAAGCGTCATTGTTTTT
	Tet-shMEIS1-R	AATTAAAAACAATGACGCTTTAAAGAGACTCGAGTCTCTTTAAAGCGTCATTG
APCDD1	Tet-shAPCDD1-F	CCGGGCTCAAACATCTCCACAATCTCGAGATTGTGGAGATGTTTGAGCTTTTT
	Tet-shAPCDD1-R	AATTAAAAAGCTCAAACATCTCCACAATCTCGAGATTGTGGAGATGTTTGAGC

Supplementary Table S5

List of primers used for luciferase reporter vector construction

Primers	Sequence (5'→3')
MEIS1-NC-F	TGGGGGAAAGTTGCAAGACCA
MEIS1-NC-R	ACTCCCCCACATCCTAGCCA
MEIS1-E1-F	TCCCCCTCACGCCTTATTGC
MEIS1-E1-R	AAGGGAGCCAGTTCCGACAC
MEIS1-E2-F	AGTGTCGGAACTGGCTCCCT
MEIS1-E2-R	AGAGCTGTGGTCTCTGGGCA
MEIS1-E3-F	TGAAGTCGAAGGATTCTGAA
MEIS1-E3-R	GACCTGCCCGCCCACGCACT
MEIS1-E4-F	AGGCACCTCCTGGGTGCAGG
MEIS1-E4-R	GTTTGCAGAGCTTTTAAAGT
MEIS1-E5-F	GAGAGGCGTGGAACTGGGTA
MEIS1-E5-R	CCTCTCCCAGCCATCAGCTG
MEIS1-E6-F	ATTACGCGAGCAATGATCTT
MEIS1-E6-R	GAAAGAGTACTGAGATGTGA
MEIS1-E7-F	ACCCCCAGCAGTCGCCTGCT
MEIS1-E7-R	ACACAAGGCATTTCGTTATT
APCDD1-NC-F	GGTGTGGAATGCATCAACTGCT
APCDD1-NC-R	AATTATCTGAGCCATTAATCAG
APCDD1-E2-F	ATCCAATTAAGTGGGGGGGGCTTA
APCDD1-E2-R	GAAGGAAGGAAAGAAGACAGGAA
APCDD1-E3-F	TCCTTCCTTCCTTCCTTCCTTCCT
APCDD1-E3-R	AGGGAGGCAGGGCCACAGTTGAGA
APCDD1-E4-F	GGCTCATTCTTTCAGGGATGGAAT

APCDD1-E4-R	AGGTACTCCCAAAGCCCGGTGCTTT
APCDD1-E5-F	ACAGCTCATTATATTTCTTGAC
APCDD1-E5-R	CTTTAAGAGAAAAGACTTTAAG

Supplementary Table S6

Super-enhancer-associated genes in at least 3 out of 4 Ewing sarcoma cell lines and 1 out of 3 primary samples

CAV2	MYL12B	CPEB2	MKNK2	SEPT9	LINGO1
FEZF1	SAMD11	HMCN1	ITGB2	SERTAD1	PRKCB
MYO10	NFIC	STMN4	CITED2	FTH1	ABHD6
TES	SUMO1P1	DLG2	ZFP36L1	PDCD6IP	CPB2
FLRT2	DIAPH3	ZDHHC3	BRD2	PAPPA	CDH11
KCNE4	LY86	LONRF1	LOC100133985	SRSF7	KLF6
AKAP7	SMAD7	TNRC18	SPEN	CHN2	ZNF532
HS3ST4	TRAPPC3	ZEB2	PPP2R2A	ACTG1	FAM84B
SLC7A11	CTDSP2	BCL11B	IRF2BP2	EPC1	PDSS1
SSH1	CRIP2	NR2F2	NCOR2	ARID1A	BMF
DPYSL2	HTT	CDC25B	FOS	ZC3H4	STEAP2
SH2B3	ZIC5	ID2	MYC	CLEC11A	MEIS1
PDE4DIP	FZD4	ZNF704	HNRNPF	TRPM4	LTBP4
RAPGEF2	UNC5B	CAPRIN1	HSPA8	FIZ1	MAP3K10
DLGAP1	TNPO2	DNAJC12	FZD1	ATP5G2	SLCO5A1
KIFC3	IKZF2	SNX18	BCL9L	CDKN2C	OR4E2
ATF7IP	ZBTB7B	GLG1	PTMA	SIRPA	RCC1
MNT	HNRNPD	PRNP	ANP32A	ARID2	PTCH1
VAX1	STK40	RERE	LMNA	MEPCE	TAS2R16
CHD9	ATP1A1	TGIF1	ADRA1D	MIR4309	MAP3K12
DAD1	CCND1	DAPK1	PCDHGC5	IGF2BP1	NFIB
KIRREL	JAK1	TMEM105	CSPG5	LIPI	NCKAP5
ZC3H13	STARD13	IER2	FADS2	HOOK1	
FCGRT	JAKMIP2	NKX2-2	KDSR	APCDD1	
ZC3H3	IRS2	RHOH	H2AFY2	PTPN13	
